# Metabolomic Abnormalities in Serum from Untreated and Treated Dogs with Hyper- and Hypoadrenocorticism

**DOI:** 10.3390/metabo12040339

**Published:** 2022-04-09

**Authors:** Carolin Anna Imbery, Frank Dieterle, Claudia Ottka, Corinna Weber, Götz Schlotterbeck, Elisabeth Müller, Hannes Lohi, Urs Giger

**Affiliations:** 1 Vetsuisse Faculty, University of Zürich, 8057 Zürich, Switzerland; carolinanna.imbery@uzh.ch; 2 Laboklin GmbH & Co. KG, 97688 Bayern, Germany; weber@laboklin.com (C.W.); mueller@laboklin.com (E.M.); 3 Institute for Chemistry and Bioanalytics, School of Life Sciences, University of Applied Sciences Northwestern Switzerland, 4132 Muttenz, Switzerland; fd@frank-dieterle.de (F.D.); goetz.schlotterbeck@fhnw.ch (G.S.); 4 PetMeta Labs Oy, 00300 Helsinki, Finland; claudia.ottka@petmetalabs.com (C.O.); hannes.lohi@petmetalabs.com (H.L.); 5 Department of Veterinary Biosciences and Department of Medical and Clinical Genetics, University of Helsinki, 00100 Helsinki, Finland; 6 Folkhälsan Research Center, 00250 Helsinki, Finland; 7 Section of Medical Genetics, University of Pennsylvania, Philadelphia, PA 19104, USA

**Keywords:** Cushing’s syndrome, Morbus Addison, canine, nuclear magnetic resonance, laboratory diagnostics, endocrinopathy

## Abstract

The adrenal glands play a major role in metabolic processes, and both excess and insufficient serum cortisol concentrations can lead to serious metabolic consequences. Hyper- and hypoadrenocorticism represent a diagnostic and therapeutic challenge. Serum samples from dogs with untreated hyperadrenocorticism (*n* = 27), hyperadrenocorticism undergoing treatment (*n* = 28), as well as with untreated (*n* = 35) and treated hypoadrenocorticism (*n* = 23) were analyzed and compared to apparently healthy dogs (*n* = 40). A validated targeted proton nuclear magnetic resonance (^1^H NMR) platform was used to quantify 123 parameters. Principal component analysis separated the untreated endocrinopathies. The serum samples of dogs with untreated endocrinopathies showed various metabolic abnormalities with often contrasting results particularly in serum concentrations of fatty acids, and high- and low-density lipoproteins and their constituents, which were predominantly increased in hyperadrenocorticism and decreased in hypoadrenocorticism, while amino acid concentrations changed in various directions. Many observed serum metabolic abnormalities tended to normalize with medical treatment, but normalization was incomplete when compared to levels in apparently healthy dogs. Application of machine learning models based on the metabolomics data showed good classification, with misclassifications primarily observed in treated groups. Characterization of metabolic changes enhances our understanding of these endocrinopathies. Further assessment of the recognized incomplete reversal of metabolic alterations during medical treatment may improve disease management.

## 1. Introduction

Gluco- and mineralocorticoids, synthesized by the adrenal cortex, play an important role in homeostasis of glucose, protein, and fat metabolism, enabling an appropriate stress response, and maintaining blood pressure and electrolyte balance [1,2]. Corticosteroid imbalances can lead to serious health problems in humans and animals, including dogs [3,4,5,6]. Hyperadrenocorticism or Cushing’s syndrome reflects a chronic excess of glucocorticoids caused by adrenal cortex or pituitary neoplasia or can develop iatrogenically by administration of glucocorticoids [6,7]. Varied clinical signs are associated with hyperadrenocorticism and hormonal and imaging tests are applied diagnostically [6,7]. Depending on the cause, the treatment may involve surgical intervention or medical treatment [6,7] with drugs such as the synthetic steroidogenesis inhibitor trilostane, which is frequently used in dogs [7]. 

In contrast, hypoadrenocorticism or adrenal insufficiency or Morbus Addison refers to a deficiency of glucocorticoids with or without lack of mineralocorticoids that results from various defects in the adrenal axis [5,8]. The clinical signs of hypoadrenocorticism vary greatly from mild unspecific signs to life-threatening adrenal crisis, and the diagnosis is based on hormonal testing [5,8]. Hormonal replacement is the mainstay of long-term treatment, while supportive therapy is necessary for cases of emergency [5,8,9].

Specific hormonal and metabolic changes have been investigated in both endocrinopathies [5,6,7,8,10]. However, comprehensive assessments of the global serum metabolomes of patients suffering from hyper- or hypoadrenocorticism are rare in any species [11,12,13,14,15,16].

Various technologies have been introduced to assess the metabolome in biological samples, such as serum, including NMR spectroscopy and mass spectrometry (e.g., gas chromatography–mass spectrometry (MS), liquid chromatography–MS) and enable the identification and quantification of large numbers of metabolites [17,18].

Here, we applied a validated ^1^H NMR spectroscopy method optimized for dogs to characterize the serum metabolomes of untreated and treated dogs with hyper- and hypoadrenocorticism. We hypothesize that (1) specific metabolic abnormalities will differentiate between hyperadrenocorticism, hypoadrenocorticism, and control dogs, (2) machine learning models using solely the serum metabolomics data will correctly differentiate both endocrinopathies and distinguish treated dogs from untreated and control dogs, and (3) medical treatment of either endocrinopathy will lead to partial or complete reversal of the metabolic abnormalities.

## 2. Results

### 2.1. Samples, Demographics, and Serum Cortisol Test Results

The serum samples selected fulfilled the entry criteria for the respective group of adult dogs with either high cortisol concentrations by low-dose dexamethasone suppression tests (LDDST) (hyperadrenocorticism untreated (HYPER_U_) group), or low cortisol concentrations by adrenocorticotropic hormone stimulation tests (ACTH-ST) (hypoadrenocorticism untreated (HYPO_U_) group), or were obtained from dogs with treated hyperadrenocorticism with low cortisol concentrations by ACTH-ST (hyperadrenocorticism treated (HYPER_T_) group; all unpaired samples with the HYPER_U_ group) (Table 1), or with treated hypoadrenocorticism (hypoadrenocorticism treated (HYPO_T_) group; 23 paired samples with the HYPO_U_ group), or from adult dogs with serum chemistry and complete blood count (CBC) results within reference intervals and thus no laboratory evidence of diseases (control (CONT) group).

A total of 153 left-over canine serum samples were included and analyzed by ^1^H NMR spectroscopy, including 27 serum samples in the HYPER_U_, 28 in the HYPER_T_, 35 in the HYPO_U_, 23 in the HYPO_T_, and 40 in the CONT group. Serum samples were mostly from Germany (89%) and rarely from other European countries (Luxembourg, Czech Republic, Romania, Finland, Norway, and Sweden). The dogs in both HYPER groups were significantly older than the dogs in the HYPO_U_ and CONT groups, albeit the age ranges were large and overlapped (Table 1). The true effect of breed could not be statistically evaluated, due to the low number of dogs per breed and group. Mixed breed dogs accounted for the largest proportion enrolled in any group. The only breed overrepresented were Dachshunds with seven and two dogs in the HYPER_U_ and HYPER_T_ groups, respectively. No differences in sex or neutering status were observed between the groups (Table 1).

### 2.2. Metabolomic Analyses

In the present study, all 105 serum metabolites assessed in the validation study for canine samples [19] were identifiable and measurable by ^1^H NMR analysis. Furthermore, 11 relative concentrations of fatty acids and seven selected amino acid ratios were calculated. The resulting 123 metabolic parameters were also documented in our recent study of canine hepatopathies [20]. Medians for all parameters of the CONT group fell in the previously established serum reference intervals for dogs of all ages [19]. However, for a few parameters, 25th or 75th percentiles of the CONT group fell slightly below (concentrations of citrate, glutamine, glycoprotein acetyls (GlycA), and large very-low-density lipoprotein (L-VLDL)-triglycerides) or slightly above (concentrations of glycine and high-density lipoprotein (HDL) particle size) the published serum reference intervals (Supplementary Table S1) [19].

No metabolomic differences were observed between two age-dependent CONT sub-groups (dogs < 6 years (yrs) old vs. dogs ≥ 6 yrs old) by univariate testing and by PCA of their serum metabolomics data which demonstrated complete overlap between the two clusters (Supplementary Figure S1). Thus, all control dogs were combined to one CONT group in the subsequent bioinformatic analyses of the metabolomics data.

#### 2.2.1. Metabolomic Comparison of HYPER_U_, HYPO_U_, and CONT Groups

**Figure 1:**
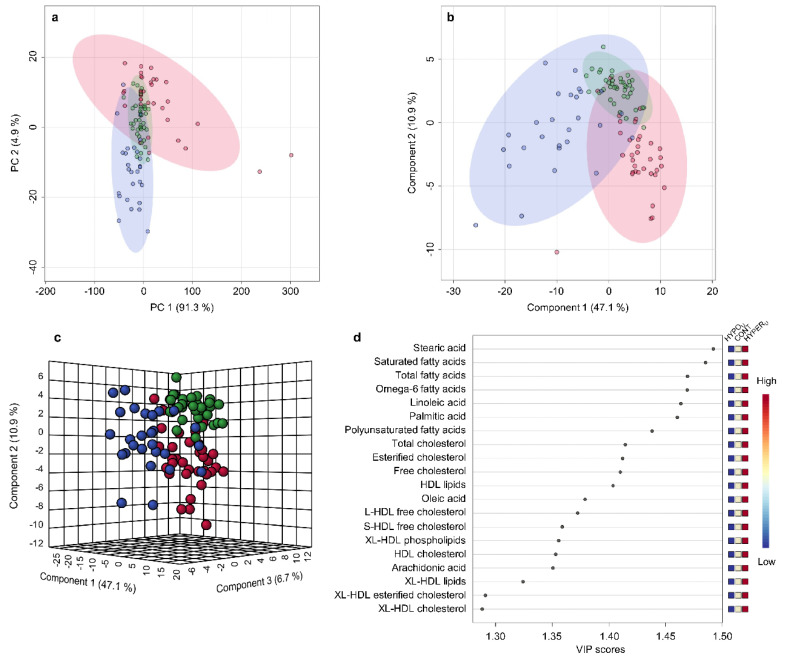
Metabolomic analyses of serum samples from dogs in the groups of hyperadrenocorticism untreated (HYPER_U_, *n* = 27), hypoadrenocorticism untreated (HYPO_U_, *n* = 35), and the control group (CONT, *n* = 40). (**a**) Scores plots of principal component analysis (PCA) and (**b**) partial least squares–discriminant analysis (PLS-DA) based on metabolomics data of serum samples of dogs in HYPER_U_ (blue), HYPO_U_ (red), and CONT (green) groups. Shaded circles represent 95% confidence intervals, while colored dots illustrate individual samples. The axes are labeled by the first (principal) components with the percentages of variance of the data explained by that component in parentheses. (**c**) The 3D scores plot of PLS-DA based on metabolomics data of serum samples of dogs in HYPER_U_ (blue), HYPO_U_ (red), and CONT (green) groups. (**d**) Variable importance in projection (VIP) scores of component 1 of the PLS-DA identifying the top 20 discriminating parameters in descending order of importance. The colored legend on the right indicates the relative abundance of the variables, with red and blue indicating high and low values, respectively, while beige illustrates neutral values.

Univariate testing of serum metabolomics data of the unpaired groups showed significant differences in 108 of 123 parameters between dogs of the HYPER_U_, HYPO_U_, and/or the CONT groups in post-hoc analyses (Supplementary Table S1). In the principal component analysis (PCA) clustering was observed in canine serum samples from the HYPER_U_, HYPO_U_, and CONT groups. While the tight cluster from the CONT group resided within the other clusters, the broader clusters of the HYPER_U_ and HYPO_U_ groups were partially distinct, and clusters extended in different directions (principal component (PC) 1 = 91.3% and PC 2 = 4.9% of total variance; Figure 1a). Many variables were found to influence the projection and separation of the groups, as reflected in marginalized parameters in the PCA loadings plot, showing relative contributions and the relationships between the parameters (Supplementary Figure S2a).

To maximize the separation of the groups, partial least squares–discriminant analysis (PLS-DA) was applied. In the PLS-DA model, the first two components contributed 58.0% of the total variance (component 1 = 47.1%, component 2 = 10.9%). As for the PCA, there was clustering of the three groups, with clusters extending into different directions. Again, the CONT group was most tightly clustered and overlapped with the broader clusters of both diseased groups (Figure 1b). Adding the third component, which contributed 6.7% of total variance, to create a 3D PLS-DA scores plot also showed tight clustering for the CONT group, while both clusters of the adrenal-diseased samples extended into different dimensions (Figure 1c). The loadings plot for the PLS-DA model is shown in Supplementary Figure S3. The PLS-DA model was validated by a 10-fold cross-validation with R^2^, Q^2^, and accuracy as displayed in Supplementary Figure S4. All figures show a robust model with three components being selected as the optimal number of components based on the Q^2^ criterion. Furthermore, a permutation test with 2,000 permutations was performed, which shows that the model is not overfitting the data (Supplementary Figure S5).

The top 20 metabolites that discriminated between the three groups were identified by the variable importance in projection (VIP) scores of the first component of PLS-DA and included many lipid-associated parameters, such as total, free, and esterified cholesterol, various HDL-associated lipid fractions, and fatty acid concentrations (Figure 1d). The first component of PLS-DA predominantly discriminated between HYPER_U_ and HYPO_U_ groups, as those mainly varied on the x-axis.

**Figure 2:**
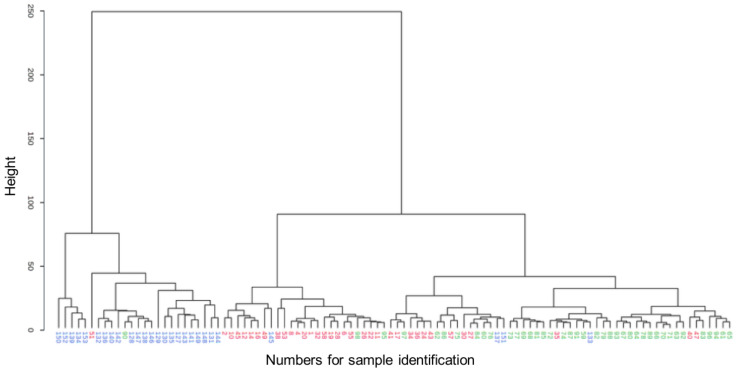
Dendrogram of hierarchical cluster analysis of serum metabolomics data from canine samples of either hyperadrenocorticism untreated (HYPER_U_, blue, *n* = 27), hypoadrenocorticism untreated (HYPO_U_, red, *n* = 35), and control (CONT, green, *n* = 40) groups. Each number on the x-axis reflects one serum sample. The y-axis shows the similarity levels expressed as Euclidean distances. Horizontal and vertical lines depict clustering of samples and differences in the distances, respectively.

Hierarchical cluster analysis of samples from dogs with untreated endocrinopathies and the CONT group revealed three main clusters and excellent separation between the groups with few exceptions. Some samples from the HYPO_U_ and CONT groups overlapped and were assigned to a cluster predominantly containing samples from the CONT group. The samples in the HYPER_U_ cluster were more distant, indicating a more different serum metabolomic profile from the other two clusters (Figure 2).

**Figure 3:**
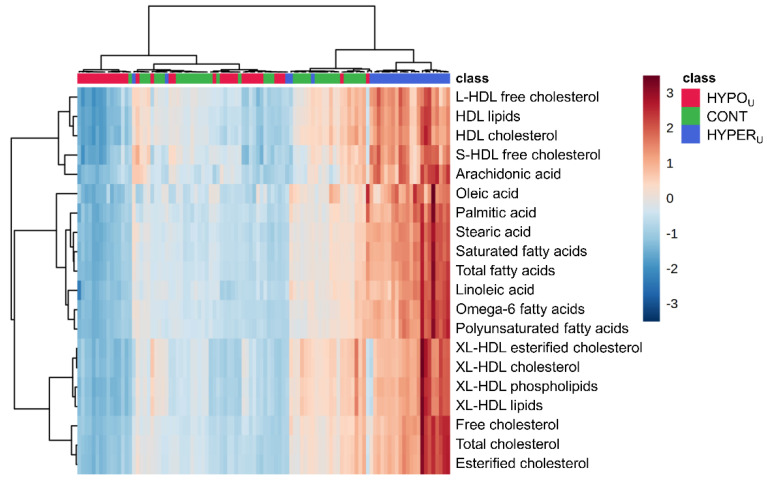
Hierarchical cluster heatmap (for samples and variables) of serum metabolomics data of canine samples in hyperadrenocorticism untreated (HYPER_U_, *n* = 27, blue), hypoadrenocorticism untreated (HYPO_U_, *n* = 35, red), and control (CONT, *n* = 40, green) groups. The top 20 parameters identified by partial least squares–discriminant analysis (PLS-DA) variable importance in projection (VIP) scores of component 1 were used. Each column represents one serum sample with group markings colored at the top. The colored legend on the right indicates the relative metabolite concentrations with different red and blue intensities indicating high and low values, respectively. Horizontal and vertical black lines depict clustering of samples and parameters.

A hierarchical cluster heat map of the top 20 parameters from PLS-DA VIP scores of the first component largely revealed higher serum metabolite concentrations in the HYPER_U_ group and lower metabolite concentrations in the HYPO_U_ group. The hierarchical cluster analysis of the heatmap assigned the samples from HYPER_U_ and HYPO_U_ groups into two clusters with some exceptions. The samples from the CONT group did not form a separate cluster but were rather distributed among those two clusters. Despite being split by some CONT samples, two subclusters mainly consisting of HYPO_U_ samples showed similarity based on color intensity patterns (Figure 3).

Machine learning methods using solely the metabolomics data were capable of correctly classifying samples into either of the untreated endocrinopathies or the CONT group in most cases (78–88%, Supplementary Table S2). Thus, with the simple logistic regression model, 88% of the samples could be assigned to the correct groups (Table 2 and Table S3, Supplementary Equation S1).

**Figure 4:**
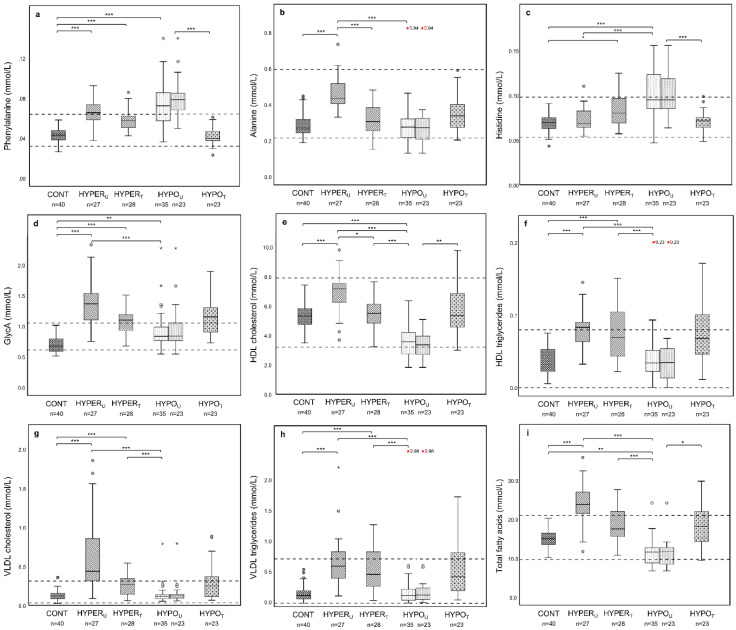
Concentrations (mmol/L) of phenylalanine (**a**), alanine (**b**), histidine (**c**), glycoprotein acetyls (GlycA) (**d**), high-density lipoproteins (HDL) cholesterol (**e**), HDL triglycerides (**f**), very-low-density lipoproteins (VLDL) cholesterol (**g**), VLDL triglycerides (**h**), and total fatty acids (**i**) in samples from dogs in the groups of CONT, HYPER_U_, HYPER_T_, HYPO_U_, and HYPO_T_. The boxes of the HYPO_U_ group are presented both from the unpaired group utilized in multivariate analyses (*n* = 35, left) and the paired group (*n* = 23, right) utilized in comparison of HYPO_U_ and HYPO_T_ groups. Boxes indicate the lower to upper quartile (25th–75th percentile) and median value. Whiskers extend to minimum and maximum values. Outliers are shown as individual open circles or stars. Dashed lines indicate reference intervals. Lines above figures reflect significant differences between specific groups (* *p* < 0.05; ** *p* < 0.01; *** *p* < 0.001). Note: CONT—control group; HYPO_U_—hypoadrenocorticism untreated; HYPO_T_—hypoadrenocorticism treated; HYPER_U_—hyperadrenocorticism untreated; HYPER_T_—hyperadrenocorticism treated.

Among the nine serum amino acids measured, phenylalanine concentrations were elevated in both endocrinopathies (Figure 4a). The HYPER_U_ group showed increased serum concentrations of tyrosine, alanine, total branched-chain amino acids (BCAA), isoleucine, and valine (Figure 4b, Supplementary Table S1), while histidine concentrations were only elevated in the HYPO_U_ group (Figure 4c). However, only slight changes in serum concentrations were observed for glycolytic metabolites, with lactate and pyruvate concentrations slightly increased in the HYPER_U_ group, and acetate and citrate concentrations slightly increased in both endocrinopathies. The concentrations of GlycA were markedly increased in the HYPER_U_ group, but only slightly increased in the HYPO_U_ group (Figure 4d).

Serum concentrations of total cholesterol (as well as concentrations of free and esterified cholesterol) were increased in the HYPER_U_ group and decreased in the HYPO_U_ group, whereas total triglyceride concentrations were only increased in the HYPER_U_ group compared to the CONT group. Concentrations of HDL and small low-density lipoprotein (LDL) particles and most of their associated lipids followed the pattern for total cholesterol concentrations for both endocrinopathies (Figure 4e,f). Concentrations of large-LDL and VLDL particles and most of their associated lipids were only increased in the HYPER_U_ group (Figure 4g,h).

Similarly, the absolute serum concentrations of total and specific fatty acids were mostly increased in the HYPER_U_ group and decreased in the HYPO_U_ group (Figure 4i). Among the relative fatty acid concentrations, palmitic acid was increased, while linoleic acid was slightly decreased in the HYPO_U_ group. Relative serum concentrations of docosahexaenoic acid were decreased in HYPER_U_ and increased in the HYPO_U_ group (Supplementary Table S1).

#### 2.2.2. Metabolomic Comparison of HYPER_U_, HYPER_T_, and CONT Groups

As the metabolomic bioinformatic data analyses described above compared HYPER_U_, HYPO_U_, and CONT groups, we next compared the serum metabolomic patterns and abnormalities in untreated and treated dogs with hyperadrenocorticism.

**Figure 5:**
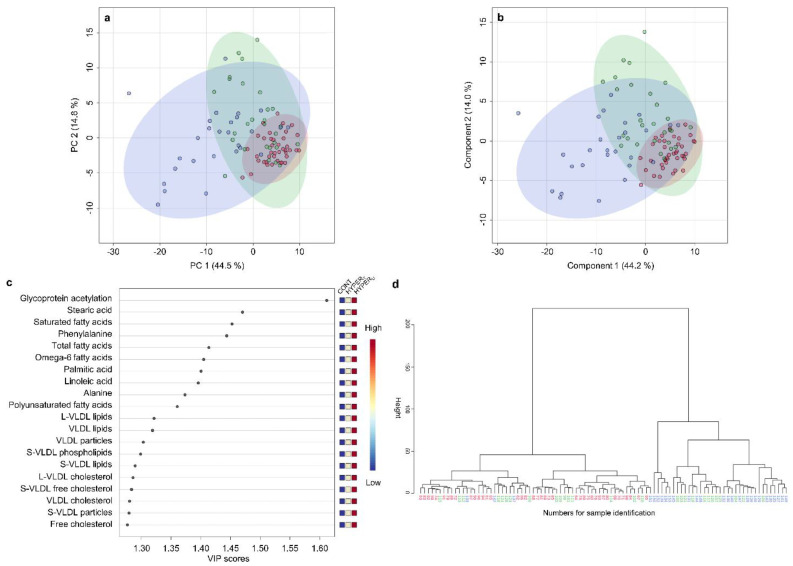
Metabolomic analyses of serum samples from dogs in the groups hyperadrenocorticism untreated (HYPER_U_, *n* = 27), hyperadrenocorticism treated (HYPER_T_, *n* = 28), and control (CONT, *n* = 40). (**a**) Scores plots of principal component analysis (PCA) and (**b**) partial least squares-discriminant analysis (PLS-DA) based on metabolomics data between serum samples of dogs in HYPER_U_ (blue), HYPER_T_ (green), and CONT groups (red). Shaded circles represent 95% confidence intervals, while colored dots illustrate individual samples. The axes are labeled by the first and second (principal) components with the percentages of variance of the data explained by that component in parentheses. (**c**) Variable importance in projection (VIP) scores of component 1 of the PLS-DA identifies the top 20 discriminating parameters in descending order of importance. The colored legend on the right indicates the relative abundance of variables, with red and blue indicating high and low values, respectively, while beige illustrates neutral values. (**d**) Dendrogram of hierarchical cluster analysis of serum metabolomic results from canine samples in either HYPER_U_ (blue), HYPER_T_ (green), or the CONT group (red). Each number on the x-axis reflects one serum sample. The y-axis shows the similarity levels expressed as Euclidean distances. Horizontal and vertical lines depict clustering of samples and differences in the distances, respectively.

In both, PCA and PLS-DA scores plots, the broad clusters of the HYPER_U_ group mostly entirely overlapped the clusters of the CONT group and the HYPER_T_ group clusters did so as well. However, even though clusters of the HYPER_U_ and HYPER_T_ groups were partly overlapping, they trended into different directions (Figure 5a,b). Total variance explained by PC 1 and 2 of the PCA model added up to 59.3%, very similar to the total variance contributed by the first two PLS-DA components, which added up to 58.2% (Figure 5a,b). According to the PCA loading plots, the separation of HYPER_U_ and HYPER_T_ groups is mostly due to elevated absolute fatty acid and lipid concentrations in the HYPER_U_ group, while in the HYPER_T_ groups relative concentrations of saturated fatty acids were increased, in addition to other parameters (Supplementary Figure S2b).

The top 20 parameters identified by the first component of PLS-DA VIP scores were mainly elevated in HYPER_U_ group and low in the CONT group compared to the HYPER_T_ group. The most discriminating measurands included GlycA, phenylalanine, alanine, multiple fatty acids, several VLDL particles and associated lipids (Figure 5c). In the hierarchical cluster analysis, samples from the HYPER_U_ and CONT groups clearly separated into two main clusters with only a few outliers, while the samples from the HYPER_T_ group were dispersed throughout the dendrogram (Figure 5d).

Various machine learning methods classified 70–88% of samples to the correct group based on the serum metabolomics data alone (Supplementary Table S2). As such, the simple logistic regression model was capable of assigning 88% of the samples correctly (Table 2b).

Most increased serum amino acid concentrations in the HYPER_U_ group were normalized in the HYPER_T_ group (alanine, total BCAA, isoleucine, valine, and tyrosine), except phenylalanine concentrations, which decreased but were still higher compared to the CONT group (Figure 4a,b). Moreover, the serum histidine concentrations, which were unchanged in HYPER_U_ dogs, actually increased in the HYPER_T_ group compared to the CONT group (Figure 4c). Increased concentrations of lactate, pyruvate, and citrate in the HYPER_U_ group normalized in the HYPER_T_ group, except for serum acetate concentrations which were still elevated compared to the CONT group (Supplementary Table S1). 

Serum GlycA concentrations diminished in samples from the treated compared to the HYPER_U_ group but remained increased (Figure 4d).

While total cholesterol concentrations normalized in the HYPER_T_ group, concentrations of total triglycerides and most triglyceride subtypes remained elevated, except for large and small LDL triglyceride concentrations, which were not altered in either the HYPER_U_ or HYPER_T_ groups. Similarly, concentrations of extra-large, large, and small VLDL particles and associated lipids, with few exceptions, remained increased in the HYPER_T_ group compared to the CONT group. However, the concentrations of most other lipoprotein particles and their associated lipid fractions (except triglyceride fractions) decreased in the HYPER_T_ group (Supplementary Table S1).

Total and most individual fatty acid concentrations tended to decrease but some remained elevated in the HYPER_T_ group (Figure 4i). Relative concentrations of fatty acids were mostly unchanged between HYPER_U_ and HYPER_T_ samples, except there was an increased relative concentration of palmitic acid in the HYPER_T_ compared to the HYPER_U_ group (Supplementary Table S1).

#### 2.2.3. Metabolomic Comparison of HYPO_U_, HYPO_T_, and CONT Groups

The serum metabolomic patterns and abnormalities in untreated and treated dogs with hypoadrenocorticism are compared below. However, while multivariate analyses and the Kruskal–Wallis test were carried out using all collected HYPO_U_ samples (*n* = 35), for the Wilcoxon signed-rank test only the paired HYPO_U_ and HYPO_T_ samples (*n* = 23) were included. 

**Figure 6:**
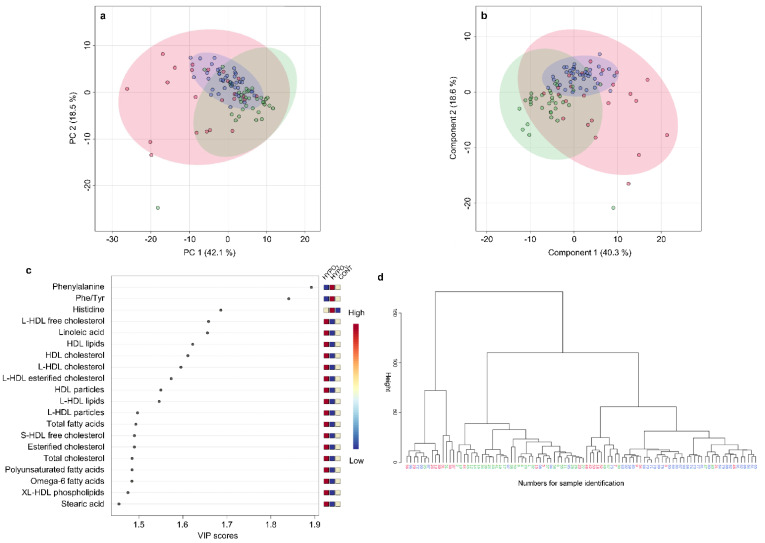
Metabolomic analyses of serum samples in the groups of hypoadrenocorticism untreated (HYPO_U_, *n* = 35), hyperadrenocorticism treated (HYPO_T_, *n* = 23), and the control group (CONT, *n* = 40). (**a**) Scores plots of principal component analysis (PCA) and (**b**) partial least squares-discriminant analysis (PLS-DA) based on metabolomics data of serum samples from dogs in HYPO_U_ (green), HYPO_T_ (red), and the CONT groups (blue). Shaded circles represent 95% confidence intervals, while colored dots illustrate individual samples. The axes are labeled by the first and second (principal) components with the percentages of variance of the data explained by that component in parentheses. (**c**) Variable importance in projection (VIP) scores of component 1 of the PLS-DA identifies the top 20 discriminating parameters in descending order of importance. The colored legend on the right indicates the relative abundance of variables, with red and blue indicating high and low values, respectively, while beige illustrates neutral values. (**d**) Dendrogram of hierarchical cluster analysis of serum metabolomic results from canine samples in either HYPO_U_ (green), HYPO_T_ (red), or the CONT group (blue). Each number on the x-axis reflects one serum sample. The y-axis shows the similarity levels expressed as Euclidean distances. Horizontal and vertical lines depict clustering of samples and differences in the distances, respectively.

In the PCA, considerable overlap of HYPO_U_, HYPO_T_, and CONT group clusters was observed (PC 1 = 42.1%, and PC 2 = 18.5% of total variance). Approximately half of the HYPO_T_ samples overlapped with the CONT group cluster, while the others spread to the left or/and downwards, causing a large HYPO_T_ cluster (Figure 6a). This shift is mainly due to the contribution of fatty acid, lipid, and lipoprotein concentrations, as shown in the PCA loadings plot (Supplementary Figure S2c). A similar clustering was seen in the PLS-DA scores plot (Figure 6b). In addition to phenylalanine and histidine, within the top 20 parameters identified by PLS-DA VIP scores of the first component, fatty acids, cholesterol subtypes, and HDL fractions were most relevant for separation (Figure 6c).

In the hierarchical cluster analysis, one large cluster consisting mainly of CONT samples formed, while samples of the HYPO_U_ group were mainly assigned to another cluster. One smaller but very distinct cluster consisted of samples from all three groups. The samples from the HYPO_T_ group were dispersed among all clusters (Figure 6d). 

Machine learning methods could correctly classify 63–78% of the samples based solely on the serum metabolomics data from the respective groups (Supplementary Table S2). To that end, 78% of the samples were assigned to the correct group in the simple logistic regression model (Table 2c). 

The increased serum phenylalanine and histidine concentrations in the group with hypoadrenocorticism decreased with treatment (Figure 4a,c). The slightly increased acetate and citrate concentrations seen in the HYPO_U_ group tended to decrease with treatment, albeit changes were not significant. The slightly increased serum GlycA concentrations in HYPO_U_ dogs further rose during treatment; also here the increase was not significant (Figure 4d, Supplementary Table S1). 

The slightly lower total serum cholesterol concentrations in the HYPO_U_ group increased in the HYPO_T_ group, whereas the increase in triglyceride concentrations in the HYPO_T_ group was not found significant. Decreased concentrations of small HDL, large HDL, and extra-large HDL particles and their associated lipid fractions in the HYPO_U_ group rose in the HYPO_T_ group (except small HDL triglyceride concentrations). While most lipid-associated parameters were within the reference intervals in the HYPO_T_ group, various LDL- and VLDL-associated lipids and triglyceride subtypes had the upper percentile slightly above the reference intervals, even if their increase was not significant compared to the HYPO_U_ group (Figure 4g,h, Supplementary Table S1). 

Likewise, the serum concentrations of total and most individual fatty acids increased with treatment of hypoadrenocorticism (Figure 4i), except for docosahexaenoic acid, which was not altered in any group, as well as concentrations of arachidonic and docosapentaenoic acid. Changes in relative fatty acid concentrations in the HYPO_T_ group compared to the HYPO_U_ group were minor and included increased relative concentrations of linoleic acid in the HYPO_T_ group and decreased relative concentrations of docosahexaenoic acid (Supplementary Table S1).

## 3. Discussion

Hypoadrenocorticism, also referred to as adrenal insufficiency, and hyperadrenocorticism, or Cushing’s syndrome, can be caused by various disorders within the pituitary–adrenal axis or can arise iatrogenically by medical or surgical interventions [5,6,7,8]. While diagnoses of these endocrinopathies are primarily based upon hormonal testing, identification of various abnormalities in routine blood tests and imaging results can further delineate cause, severity, and complications [5,6,7,8]. In addition, these tests can be used to clinically monitor and adjust therapeutic interventions for hypo- and hyperadrenocorticism [5,6,7,8].

It is well recognized that endogenous and exogenous glucocorticoid imbalances can lead to profound metabolic dysfunction with varied severity of systemic illnesses in humans and animals [5,6,7,8]. While such dysfunction is expected to have a major impact on the metabolome, there is currently a paucity of data on the impact of excess or deficient corticosteroid levels on the serum metabolome of humans and dogs with these adrenal endocrinopathies, either untreated or during treatment [11,12,13,14,15,16]. 

Utilizing a validated ^1^H NMR method for dogs, we assessed serum samples from untreated and treated dogs with hyper- and hypoadrenocorticism. Our findings provide the first evidence of often contrasting metabolite abnormalities and distinct metabolomic patterns in these canine adrenal endocrinopathies and reveal that these metabolic changes do not completely return to baseline with treatment in either condition. The metabolomic changes reported here will most probably not replace the current diagnostic tests for canine adrenal endocrinopathies but suggest that this metabolomic platform has the potential to further define metabolic dysfunction in hyper- and hypoadrenocorticism and may aid in clinical monitoring to optimize medical treatment.

Using this novel ^1^H NMR spectroscopy platform, we documented major abnormalities in the serum metabolome of dogs with endogenous hyper- and hypoadrenocorticism, with 108 of 123 metabolic measurands differing between the two untreated endocrinopathies and/or the control group. As cortisol is a powerful catabolic hormone with broad metabolic effects [2], this number of abnormalities may not be surprising. Moreover, many lipid- and fatty acid-associated metabolites were increased in serum of dogs with hyperadrenocorticism and decreased with hypoadrenocorticism, highlighting the effects of cortisol on their metabolism. However, other metabolites were altered similarly in both endocrinopathies, which could either reflect different metabolic pathways affecting the same metabolites or general disease-related changes. It should be noted that all observed changes in measurands were modest, with less than three-fold differences from the control group and close to reference intervals.

Metabolomics data were analyzed by both univariate and multivariate analyses, such as PCA, PLS-DA, hierarchical cluster analyses, and machine learning methods. The clusters of the control dogs in PCA and PLS-DA were the tightest, suggesting that the broader but distinct clusters of samples from dogs with untreated hyper- and hypoadrenocorticism (Figure 1a,b, Figure 5a,b and Figure 6a,b) may be due to different disease stages, duration, or associated complications, including hypertension, inflammation, cholestatic disease, or others in dogs with endogenous hyperadrenocorticism [7,21] or hypovolemic shock in dogs with endogenous hypoadrenocorticism [8,22]. Furthermore, we did not distinguish between adrenal- and pituitary-dependent hyperadrenocorticism, which may have slightly different serum metabolomic patterns. In addition, while the great majority of dogs with endogenous hypoadrenocorticism involve both, a glucocorticoid and mineralocorticoid deficiency and can show greatly varied clinical manifestation [8,22], we did not attempt to differentiate in the present study between both, nor between primary and secondary hypoadrenocorticism, which might also contribute to the broadness of the clusters in PCA and PLS-DA (Figure 1a,b and Figure 6a,b). Finally, as seen in hierarchical cluster analysis, samples in the cluster of dogs with hyperadrenocorticism were more distant from the cluster of samples of dogs with hypoadrenocorticism and from the control group, indicating greater differences in the serum metabolome of these dogs (Figure 2).

Many metabolic abnormalities in serum lipid-associated parameters, e.g., cholesterol subtypes, HDL-associated lipids, and absolute fatty acid concentrations in samples of dogs with hyper- and hypoadrenocorticism were noted (Supplementary Table S1). Changes were often in the opposite direction, with higher and lower serum concentrations in hyper- and hypoadrenocorticism, respectively. These measurands markedly influenced the separation of cluster analyses, as reflected by PLS-DA VIP scores (Figure 1d). Lipid-associated abnormalities, including hypercholesterolemia and hypertriglyceridemia occur in humans and dogs with hyperadrenocorticism and during exogenous glucocorticoid exposure [6,7,23,24,25,26,27], and are possibly caused by glucocorticoid effects on lipolysis, free fatty acid production, and VLDL synthesis [24]. Furthermore, glucocorticoids promote cholesterol synthesis through enzyme induction in rat hepatocytes [28,29]. Dogs with untreated hyperadrenocorticism also showed increased serum cholesterol and triglyceride concentrations in this study, albeit the median values were still in the reference intervals. However, in a recent experimental lipidomic study of tetracosactide-induced hypercortisolism in dogs increased total and specific fractions of plasma triglycerides have been found, but not total cholesterol concentrations [30]. 

The VLDL and LDL cholesterol concentrations in the group with hyperadrenocorticism were markedly increased, while the increased HDL cholesterol concentrations were within the reference interval (Figure 4e,g). Additionally, we observed increases in VLDL and HDL triglyceride concentrations in the group of untreated hyperadrenocorticism. Prior observations in dogs with hyperadrenocorticism demonstrated mainly increased VLDL cholesterol fractions but decreased HDL cholesterol and triglyceride fractions (percentage distribution), and also absolute increased concentrations of VLDL cholesterol and triglycerides [23], and mainly increased LDL cholesterol concentrations [31]. Humans with Cushing’s syndrome show increased LDL and VLDL concentrations leading to hypercholesterolemia and hypertriglyceridemia [6], while HDL cholesterol concentrations were not found to be decreased [25]. 

Low serum cholesterol concentrations have previously been reported in dogs with hypoadrenocorticism [22] and based upon this study, are mainly characterized by a decrease in HDL cholesterol concentrations (Figure 4e, Supplementary Table S1). Suggested causes for the changes in cholesterol concentrations include decreased lipid absorption by the gastrointestinal tract, decreased fatty acid mobilization due to low cortisol, and increased utilization of fatty acids related to high adrenocorticotropic hormone (ACTH) concentrations [22]. As glucocorticoids induce enzymes that enhance cholesterol synthesis [28,29], their lack may contribute to low cholesterol levels. Human patients cured from Cushing’s disease exhibit persistently altered lipid markers [32,33]. Similarly, we found that while many HDL- and LDL-associated lipid changes were reversible with treatment of hyperadrenocorticism, many VLDL-associated lipids remained elevated (Supplementary Table S1). 

Dogs with treated hypoadrenocorticism also showed altered lipid and lipoprotein profiles compared to the untreated dogs. However, concentrations of triglycerides and predominantly VLDL-associated lipids tended to increase, despite being unaltered prior to treatment. Although these increases were not statistically significant, the upper percentiles of these parameters were often above the reference intervals (Figure 4g,h, Supplementary Table S1). Such finding could be related to the administration of glucocorticoids, which could lead to the secretion of VLDL and increase in triglyceride concentrations [23], and administration of fludrocortisone or fludrocortisone and prednisone also increased cholesterol and triglyceride concentrations without inducing clinical features of iatrogenic hyperadrenocorticism in treated dogs [9].

This ^1^H NMR platform determines the total concentration of free (non-esterified) and esterified fatty acids. The high and low total and specific fatty acid concentrations observed in hyper- and hypoadrenocorticism (Figure 4i, Supplementary Table S1) depend on lipid concentrations and also on the lipoprotein profile, as the different lipoproteins and their lipids are esterified with distinct fatty acids [34]. Furthermore, glucocorticoids may also increase free fatty acids [24,35] and may lead to further increased fatty acid concentrations in dogs with hyperadrenocorticism.

The ^1^H NMR platform utilized in this study only identifies nine amino acids, and the observed differences from the apparently healthy control group and canine reference interval were rather small (Supplementary Table S1). Nevertheless, the increase in serum alanine concentrations in canine hyperadrenocorticism in our study (Figure 4b) was similar to that in human patients with Cushing’s disease and prednisolone administration [36,37]. This was associated with reduced protein synthesis and insulin resistance, which may stimulate the glucose–alanine cycle [36]. In contrast, the increased serum BCAA, phenylalanine, and tyrosine concentrations seen in dogs with hyperadrenocorticism (Supplementary Table S1) were only partially consistent with metabolic patterns seen in human patients with Cushing’s syndrome or disease [12,13,36] and may be related to species-specific metabolic differences. Dogs with hypoadrenocorticism had increased serum phenylalanine and histidine concentrations (Figure 4a,c). The unaltered histidine concentrations in dogs with hyperadrenocorticism are in contrast to a human study that found decreased serum histidine concentrations in patients with Cushing’s syndrome [12].

Serum GlycA is an inflammatory biomarker that reflects the signals of N-acetylglucosamine residues within certain acute-phase proteins, mainly α1-acid glycoprotein, α1-antitrypsin, α1-antichymotrypsin, haptoglobin, and transferrin [38,39]. While there are no prior studies on serum GlycA concentrations in dogs with altered cortisol levels, the increased GlycA concentrations seen in dogs with hyperadrenocorticism in our study (Figure 4d), may be equated with the previously reported increased haptoglobin concentrations in canine hyperadrenocorticism [40,41,42,43]. While the GlycA concentrations fell slightly following treatment of hyperadrenocorticism, they remained increased over controls (Figure 4d). Likewise, serum haptoglobin concentrations decreased in dogs with treated hyperadrenocorticism, but were still elevated, potentially due to poor control of hyperadrenocorticism, cortisol precursors, or secondary effects of hyperadrenocorticism [40,41,44]. In human pediatric patients with Cushing’s disease, serum GlycA concentrations declined after transsphenoidal surgery [33]. We also found that serum GlycA concentrations are increased in dogs with hypoadrenocorticism (Figure 4d); however, the mechanisms responsible are unknown, and clinical implications remain unclear.

While a prior study predicted canine hyperadrenocorticism based on demographic data, clinical signs, and liver enzyme activities [45] and another predicted canine hypoadrenocorticism based on CBC and serum chemistry screening results [46], this is the first study to apply machine learning approaches based solely on metabolomics data. The applied machine learning tools performed well, predicting the correct groups when applied to the two untreated endocrinopathies and the control group (Table 2, Supplementary Table S2). However, hyper- and hypoadrenocorticism show different clinical and laboratory characteristics [7,8,10,21], as well as contrasting metabolic features. As such, future studies would be needed to compare both endocrinopathies to other clinically similar diseases to determine if this machine learning approach can correctly predict disease based on metabolomics data. Furthermore, the etiology of these endocrinopathies should be considered in future machine learning studies to improve classification. In the simple logistic regression model some samples from dogs with hypoadrenocorticism were misclassified as control samples (Table 2a), suggesting relatively minor metabolic changes as also reflected by dogs with hypoadrenocorticism often showing mild signs that make a prompt diagnosis difficult [8,10,22]. Machine learning approaches were slightly less accurate in classifying treated dogs compared to classifying either untreated endocrinopathy or controls (Table 2b,c and Table S2). A false classification of samples from treated dogs could be due to normalization of metabolic abnormalities during treatment (misclassified as control sample), or due to insufficient resolution of metabolic changes (misclassified as untreated sample). 

Although we expected normalization of metabolic changes in serum of treated dogs, this first of its kind study revealed a more complex picture of serum metabolomic changes following treatment of dogs with hyper- and hypoadrenocorticism. While some parameters regained values within the reference interval comparable to the control group, others were persistently abnormal. In fact, parameters that tended to be overcompensated in treated dogs with hypoadrenocorticism (no statistical significance), such as different types of triglycerides or VLDL-associated metabolites, only showed partial reversal in treated dogs with hyperadrenocorticism (Figure 4g,h, Supplementary Table S1). As these changes appear to be consistent with glucocorticoid effects, it suggests that some dogs may not have been treated long enough or may not have been ideally managed. However, as the duration between diagnosis and sampling for the treated endocrinopathy groups was not standardized, this might also affect the changes in the serum metabolome. Short- and long-term induced canine hypercortisolism also showed some different lipidomic alterations [30]. While the medical treatment of dogs with endogenous adrenal diseases is clinically monitored for efficacy and safety by hormonal and routine blood tests in addition to clinical signs, treatment of both endocrinopathies can be challenging [7,8]. For example, ACTH-ST results for monitoring post-trilostane cortisol levels during treatment for hyperadrenocorticism were inconsistent with clinical signs [47,48]. However, in this study, the treated hyperadrenocorticism group showed relatively low pre- and post-ACTH-ST cortisol levels, and dogs with post ACTH-ST cortisol of less than 10 ng/mL (1 µg/dL) may be overtreated [7]. Additionally, due to the use of left-over samples the time between medical treatment and sampling remains unknown and might be different. In treatment of canines with hypoadrenocorticism, dosage for mineralocorticoids can be monitored with electrolyte concentrations, but glucocorticoid dosages are adapted according to clinical signs, so over- and undertreatment may occur [8,49,50]. 

In addition to the limitations of our study mentioned above, our comparative investigation of the serum metabolomics of treated and untreated dogs with either hyper- or hypoadrenocorticism was undertaken with a relatively small number of subjects, which were not specifically sex- and age-matched, and no attempt was made to differentiate between etiology, severity, and duration of the endocrinopathy. Dogs with hyperadrenocorticism were older than those of the other groups. However, the ranges of ages in each group were broad and overlapping. In a simultaneously performed study on canine hepatopathies, we showed that there were no significant differences in serum concentrations of the metabolomics data between younger and older adult control dogs (expect citrate concentrations, *p* = 0.049) [20]. The serum samples of the CONT group used in this study were also part of the control group in this recent study on canine hepatopathies [20]. Likewise, in this study we did not identify significant differences in the metabolomics data by univariate analysis with Kruskal–Wallis test adjusted with Bonferroni correction and with PCA of the metabolomics data between two age-dependent CONT subgroups divided at the age of 6 yrs (Supplementary Figure S1). To simplify the bioinformatic analyses, we only showed the combined CONT group as we did for our metabolomic study on canine hepatopathies [20]. However, future prospective studies should consider specific breed-, sex-, and age-matching.

Similarly, exact treatment regimens (e.g., glucocorticoid +/− mineralocorticoid treatment in the HYPO_U_ group, or any other supportive care) and clinical responses to treatment were not available, and thus their impact on metabolomics data could not be ascertained. Likewise, other factors such as body condition score, diet, and time-interval of treatment were not included. The duration of treatment especially in hypoadrenocorticism might have an influence on the serum metabolome, as the dosages at the beginning of the treatment are often higher and then are tapered off [8,9]. As the groups of dogs with hyperadrenocorticism were not paired, individual influences on the serum metabolome might have been neglected. As none of the authors were attending veterinarians to any dogs of this study and left-over samples were used, occult diseases which were not detected by routine blood test results in the CONT group cannot be fully excluded. Due to the use of left-over samples the sampling was not standardized and there might be a varied lag period from sample collection and separation of serum from clot until chilling and freezing which could affect certain metabolites results. Also, feeding and fasting were not standardized. However, the metabolic abnormalities that we observed during treatment potentially identify metabolomic testing as a novel approach to guide and adjust treatment of adrenal endocrinopathies more precisely. Finally, it will need to be determined if our findings are consistent with other metabolomic platforms to further evaluate its promise in assessing disease and treatment of adrenal diseases.

## 4. Materials and Methods

### 4.1. Samples and Groups

The study was conducted using left-over serum samples submitted between May 2020 and June 2021 for routine testing to a veterinary diagnostic laboratory (Laboklin GmbH & Co. KG, Bad Kissingen, Germany). Untreated and treated adult dogs with hyper- and hypoadrenocorticism, as well as apparently healthy adult dogs were included in this study. The use of left-over samples for research purposes was approved by the government in Lower Franconia, Bavaria, Germany (RUF-55.2.2-2532-1-86-5). Laboklin’s electronic database was searched for results LDDST and ACTH-ST, supporting an adrenal disorder [8,51,52]. Only basal serum samples from LDDST or ACTH-ST were analyzed in metabolomic analyses. 

The following groups of dogs were assessed in this study:

- **HYPER_U_ group**—samples from dogs with high serum cortisol concentrations of >10 ng/mL (>1 µg/dL) in both pre- and 8 h post-LDDST samples supportive of a diagnosis of hyperadrenocorticism [51,52].
- **HYPER_T_ group**—samples from treated dogs with hyperadrenocorticism and serum cortisol concentrations of <20 ng/mL (<2 µg/dL) in pre- and post-ACTH-ST samples. All HYPER_T_ dogs were different from the HYPER_U_ dogs (unpaired samples).
- **HYPO_U_ group**—samples with low serum cortisol concentrations of <10 ng/mL (<1 µg/dL) in both pre- and post-ACTH-ST samples consistent with a diagnosis of hypoadrenocorticism [8].
- **HYPO_T_ group**—samples from dogs in the HYPO_U_ group mentioned above were examined once during treatment for at least two weeks. For those dogs, routine blood testing during treatment was offered to attending clinicians (free of charge), and the left-over serum sample was used for the metabolomic study (paired samples).
- **CONT group**—samples from adult dogs with serum chemistry panel and CBC results in the reference intervals. All serum samples from apparently healthy dogs were also part of the control group in our recent metabolomic study on canine hepatopathies [20]. No metabolomic differences were observed between two age-dependent CONT subgroups (dogs < 6 yrs old vs. dogs ≥ 6 yrs old) by univariate testing with Kruskal–Wallis test adjusted with Bonferroni correction and by PCA of their serum metabolomics data (Supplementary Figure S1). Thus, to simplify the presentation the control dogs were combined to one CONT group for bioinformatic analyses of the metabolomics data.

Available information on breed, age, sex, neutering status, and other data received from submission forms, medical consult service, as well as blood test results were gathered and reviewed. For both untreated endocrinopathies (HYPER_U_ and HYPO_U_) groups, only samples from dogs without known concurrent diseases (e.g., infectious diseases, other specific organ diseases) and for the CONT group only samples without laboratory evidence of any disease were included. Furthermore, clinical information from submission forms and from contacting the submitting veterinary clinicians by Laboklin’s medical consult service was obtained to support the diagnosis of hyper- or hypoadrenocorticism in dogs of the HYPER_U_ and HYPO_U_ groups, respectively, and to exclude other diseases and prior treatment with glucocorticoids or trilostane in the HYPO_U_ group. 

The laboratory’s inventory of frozen samples was screened for left-over serum samples with a residual volume of ≥300 µL. These serum samples were originally submitted to the laboratory after centrifugation and removal of clotted blood and were delivered either chilled or unchilled if transport time was ≤1 day. Samples with hemolysis and/or icterus were excluded. Frozen serum samples were thawed, aliquoted (1.8 mL CryoPure tubes, Sarstedt AG & Co. KG, Nürnbrecht, Germany), and refrozen at −80 °C until shipment for metabolomic analysis within ≤6 months. Results of serum chemistry analyses (Cobas 8000 c701 analyzer, Roche Diagnostics, Mannheim, Germany) and CBC (ADVIA 2120i, Siemens Healthcare GmbH, Erlangen, Germany or Sysmex XT2000i, Sysmex Deutschland GmbH, Norderstedt, Germany) were reviewed. Serum chemistry analyses were performed on thawed samples, if not already undertaken during routine testing. Cortisol measurements for LDDST and ACTH-ST were conducted with a Cobas 8000 e602 analyzer with an electrochemiluminescence immunoassay (Roche Diagnostics, Mannheim, Germany).

### 4.2. Serum Metabolomic Analyses

The metabolomic analysis of canine serum samples was performed as previously described in [19]. Serum samples were shipped frozen overnight on ice packs to PetMeta Labs Oy (Helsinki, Finland). Targeted metabolomic analysis was conducted with a ^1^H NMR spectrometer (Bruker AVANCE III HD 500 MHz, Bruker Corp., Billerica, MA, USA). The ^1^H NMR method used is optimized for dogs and validated for canine serum and plasma samples [19]. A similar ^1^H NMR method has been described and largely utilized for human serum and plasma samples [53]. Metabolomics data were reported after spectral processing as metabolite concentrations, and ratios and percentages were calculated. Unnamed peaks were not reported and thus not included in further analyses.

### 4.3. Statistical Analysis

#### 4.3.1. Univariate Analyses

Univariate statistical analyses were performed using MS Office Excel (Microsoft Corp., Redmond, WA, USA) and SPSS Statistics (version 26; IBM Corp., Armonk, NY, USA) software programs. All continuous data were assessed for normal distribution. Differences in age were evaluated using a one-way analysis of variance (ANOVA) [54]. Differences in sex and neutering status were evaluated using chi-square tests.

Concentrations of metabolomics data missing at random were imputed by the median of the corresponding variable, and concentrations below the detection limit were imputed with a zero value. Differences in metabolomics data were assessed with Kruskal–Wallis test for comparison of unpaired samples (CONT, HYPER_U_, HYPER_T_, and HYPO_U_ groups, as well as for the age-dependent CONT subgroups, HYPER_U_, HYPER_T_, and HYPO_U_ groups) [55] and with a Wilcoxon signed-rank test for the paired samples of HYPO_U_ and HYPO_T_ [56], both adjusted with a Bonferroni correction [57]. The level of significance was set at *p* < 0.05.

While in the Kruskal–Wallis test and multivariate analyses all collected HYPO_U_ samples (*n* = 35) are included, the Wilcoxon signed-rank test includes only the paired HYPO_U_ and HYPO_T_ samples (*n* = 23).

#### 4.3.2. Multivariate Analyses

Imputation of serum metabolomics data was completed as described above. PCA [58], PLS-DA [59], hierarchical cluster analyses, and the hierarchical cluster heatmap of the serum metabolomics data were performed using MetaboAnalyst 5.0 [60] with auto-scaled variables. Hierarchical cluster analyses were performed using the Ward clustering algorithm and the Euclidean distance measure [61]. A hierarchical cluster heatmap was created to visualize changes in the 20 most discriminative parameters identified by VIP scores in PLS-DA. Machine learning methods were performed with Waikato Environment for Knowledge Analysis (WEKA) 3.95 [62]. Models applied were simple logistic regression [63], support vector machines [64], k-nearest neighbors (KNN) algorithm [65], Multilayer Perceptron (MLP) Classifier [66], Random Forest [67], and multinomial naïve Bayes [68]. The default settings of the parameters for the respective WEKA implementation were used for all machine learning methods. Machine learning models were evaluated using 10-fold full cross-validation for each model.

## 5. Conclusions

Using a targeted metabolomic ^1^H NMR platform quantifying 123 metabolic parameters, this study revealed distinct metabolomic patterns and major metabolic abnormalities in the serum of dogs with untreated and treated hyper- or hypoadrenocorticism. Serum amino acid concentrations changed in various directions, with serum phenylalanine concentrations being increased in both endocrinopathies, while serum concentrations of tyrosine, alanine, and total branched-chain amino acid were only increased in hyperadrenocorticism, and histidine concentrations were elevated in hypoadrenocorticism. Various lipoprotein and lipid fractions, and fatty acid concentrations were often opposingly altered and were predominantly increased in hyperadrenocorticism and decreased in hypoadrenocorticism. These metabolic changes may give new insights in the pathophysiology and improve characterization of these endocrinopathies. It remains unclear why the metabolic alterations were only partially reversed following treatment, so further investigations are warranted to enhance our understanding of disease management. Further optimization of applied machine learning approaches may facilitate future diagnosis or improve monitoring of treatment outcomes for these patients.

## Figures and Tables

**Table 1:** Demographic data of 153 serum samples from dogs in the groups of CONT (*n* = 40), HYPER_U_ (*n* = 27), HYPER_T_ (*n* = 28), HYPO_U_ (*n* = 35), and HYPO_T_ (*n* = 23).

	CONT	HYPER_U_	HYPER_T_	HYPO_U_	*p* Value	HYPO_U_^†^	HYPO_T_^†^
**Number of dogs**, n	40	27	28	35		23	23
**Age**, years, median (range)	5.3 (1.3–11.0) ^a^	11.0 (8.0–14.0) ^b^	11.0 (6.3–15.0) ^b^	6.0 (0.8–12.0) ^a^	<0.001	6.0 (1.3–11.0)	6.0 (1.3–11.0)
**Breeds**, n							
Mixed breed	11	10	11	18		11	11
Others ^¥^/Dachshund	29/0	10/7	15/2	17/0		12/0	12/0
**Sex**, n							
Males, intact/castrated	12/9	8/7	9/4	9/12	>0.05	4/10	4/10
Females, intact/spayed	10/8 ^‡^	7/5	2/13	4/10		1/8	1/8
**Cortisol ACTH-ST**, ng/mL, median (range)							
Cortisol pre-ACTH	ND	ND	5.5 (1.4–13.8)	0.5 (0.5–4.3)		0.5 (0.5–4.3)	ND
Cortisol post-ACTH	ND	ND	12.2 (3.0–19.9)	0.5 (0.5–6.1)		0.5 (0.5–3.9)	ND
**Cortisol LDDST**, ng/mL, median (range)							
Cortisol pre-dexamethasone	ND	55.9 (15.5–300.7)	ND	ND		ND	ND
Cortisol 8 hrs post-dexamethasone	ND	36.5 (11.2–86.4)	ND	ND		ND	ND

Note. ^¥^ Breed with ≤3 dogs/breed/group. ^‡^ Sex was not reported for one dog. ^†^ Comparison of the 23 paired samples from dogs in HYPO_U_ and HYPO_T_. Results with different letter superscripts (^a, b^) in the same line are significantly different from each other. ACTH—adrenocorticotropic hormone; ACTH-ST—adrenocorticotropic hormone stimulation test; CONT—control group; hrs—hours; HYPO_U_—hypoadrenocorticism untreated; HYPO_T_—hypoadrenocorticism treated; HYPER_U_—hyperadrenocorticism untreated; HYPER_T_—hyperadrenocorticism treated; LDDST—low-dose dexamethasone suppression test; ND—not determined.

**Table 2:** Simple logistic regression model to classify dogs based on the metabolomics data into the groups of (a) HYPER_U_, HYPO_U_, and CONT, (b) HYPER_U_, HYPER_T_, and CONT, (c) HYPO_U_, HYPO_T_, and CONT compared to the clinicopathologically assigned groups.

	Clinicopathologically Assigned Groups	Dogs, n	Groups Assigned by Simple Logistic Regression Model		
**a**			**CONT**	**HYPER_U_**	**HYPO_U_**
	**CONT**	40	**38**	1	1
	** HYPER_U_**	27	1	**25**	1
	**HYPO_U_**	35	6	2	**27**
**b**			**CONT**	**HYPER_U_**	**HYPER_T_**
	**CONT**	40	**40**	0	0
	**HYPER_U_**	27	1	**24**	2
	**HYPER_T_**	28	5	3	**20**
**c**			**CONT**	**HYPO_U_**	**HYPO_T_**
	**CONT**	40	**32**	3	5
	**HYPO_U_**	35	5	**28**	2
	**HYPO_T_**	23	6	1	**16**

Note: CONT—control group; HYPO_U_—hypoadrenocorticism untreated; HYPO_T_—hypoadrenocorticism treated; HYPER_U_—hyperadrenocorticism untreated; HYPER_T_—hyperadrenocorticism treated.

## Data Availability

Data is contained within the article or supplementary material. Any further data may be requested from the corresponding author. The data are not publicly available due to it contains patient information.

## Supplementary Materials

The following supporting information can be downloaded at: https://www.mdpi.com/article/10.3390/metabo12040339/s1, Table S1: Metabolomic serum parameters significantly differing between dogs in the groups of CONT (*n* = 40), HYPER_U_ (*n* = 27), HYPER_T_ (*n* = 28), HYPO_U_ (*n* = 35), and HYPO_T_ (*n* = 23); Table S2: Different machine learning models classifying groups based solely on metabolomics data from serum samples of dogs in the groups of CONT (n = 40), HYPER_U_ (*n* = 27), and HYPO_U_ (*n* = 35); HYPER_U_ (*n* = 27), HYPER_T_ (*n* = 28), and CONT (*n* = 40); HYPO_U_ (*n* = 35), HYPO_T_ (*n* = 23), and CONT (*n* = 40); Table S3: Detailed accuracy by class for the simple logistic regression model of the metabolomics data from serum samples of dogs in the groups of CONT (*n* = 40), HYPER_U_ (*n* = 27), and HYPO_U_ (*n* = 35); Figure S1: Comparison of age and metabolomics data of CONT groups subdivided at the age of 6 years into dogs of younger (<6 yrs, *n* = 22) and of older age (≥6 yrs, *n* = 18); Figure S2: Loadings plot of principal component analysis based on metabolomics data between serum samples (a) of dogs in the groups of CONT (*n* = 40), HYPER_U_ (*n* = 27), and HYPO_U_ (*n* = 35); (b) HYPER_U_ (*n* = 27), HYPER_T_ (*n* = 28), and CONT (*n* = 40); (c) HYPO_U_ (*n* = 35), HYPO_T_ (*n* = 23), and CONT (*n* = 40); Figure S3: Loadings plot of partial least squares–discriminant analysis (PLS-DA) based on metabolomics data between serum samples of dogs in the groups of CONT (*n* = 40), HYPER_U_ (*n* = 27), and HYPO_U_ (*n* = 35); Figure S4: Results of the 10-fold cross-validation of the partial least squares–discriminant analysis (PLS-DA) model based on metabolomics data between serum samples of dogs in the groups of CONT (*n* = 40), HYPER_U_ (*n* = 27), and HYPO_U_ (*n* = 35) with R^2^, Q^2^, and accuracy measures based on the number of components; Figure S5: Results of a permutation test with 2000 permutations for the partial least squares–discriminant analysis (PLS-DA) based on metabolomics data between serum samples of dogs in the groups of CONT (*n* = 40), HYPER_U_ (*n* = 27), and HYPO_U_ (*n* = 35); Equation S1: Equation of simple logistic regression model of the metabolomics data from serum samples of dogs in the groups of CONT (*n* = 40), HYPER_U_ (*n* = 27), and HYPO_U_ (*n* = 35).
