# Supplementary material for: Metabolomic Abnormalities in Serum from Untreated and Treated Dogs with Hyper- and Hypoadrenocorticism
Source: Metabolites. 2022 Apr 9;12(4):339. doi: 10.3390/metabo12040339 (PMC9028761; doi:10.3390/metabo12040339)
Supplement: Supplementary file 1 [file metabolites-12-00339-s001.zip › metabolites-1624636-supplementary.pdf]

Metabolomic Abnormalities in Serum from Untreated and Treated Dogs with Hyper- and Hypoadrenocorticism

Carolin Anna Imbery [1,2], Frank Dieterle [3,†], Claudia Ottka [4,5,6,†], Corinna Weber [2], Götz Schlotterbeck [3], Elisabeth Müller [2], Hannes Lohi [4,5,6] and Urs Giger [1,7,*]

[1] Vetsuisse Faculty, University of Zürich, 8057 Zürich, Switzerland; carolinanna.imbery@uzh.ch
[2] Laboklin GmbH & Co. KG, 97688 Bayern, Germany; weber@laboklin.com (C.W.); mueller@laboklin.com (E.M.)
[3] Institute for Chemistry and Bioanalytics, School of Life Sciences, University of Applied Sciences Northwestern Switzerland, 4132 Muttenz, Switzerland; fd@frank-dieterle.de (F.D.); goetz.schlotterbeck@fhnw.ch (G.S.)
[4] PetMeta Labs Oy, 00300 Helsinki, Finland; claudia.ottka@petmetalabs.com (C.O.); hannes.lohi@petmetalabs.com (H.L.)
[5] Department of Veterinary Biosciences and Department of Medical and Clinical Genetics, University of Helsinki, 00100 Helsinki, Finland
[6] Folkhälsan Research Center, 00250 Helsinki, Finland
[7] Section of Medical Genetics, University of Pennsylvania, Philadelphia, PA 19104, USA
* Correspondence: giger@upenn.edu
† These authors contributed equally to this work

Supplementary Table S1 Metabolomic serum parameters differing between dogs in the groups of CONT (n=40), HYPER$_U$ (n=27), HYPER$_T$ (n=28), HYPO$_U$ (n=35), and HYPO$_T$ (n=23).

Parameter	Reference interval*	CONT‡	HYPER$_U$‡	HYPER$_T$‡	HYPO$_U$‡	*p* value‡	HYPO$_U$¥	HYPO$_T$¥	*p* value¥
**Amino acids**, median (range)									
Phenylalanine	0.03-0.07 mmol/L	0.04 (0.03-0.06)[a]	0.07 (0.04-0.09)[b]	0.06 (0.04-0.09)[b]	0.07 (0.04-0.14)[b]	<.001	0.08 (0.05-0.14)	0.04 (0.02-0.06)	<.001
Tyrosine	0.04-0.09 mmol/L	0.06 (0.04-0.09)[a]	0.08 (0.06-0.12)[b]	0.06 (0.04-0.11)[a]	0.07 (0.04-0.09)[a]	<.01	0.07 (0.04-0.09)	0.06 (0.05-0.08)	>.05
Histidine	0.05-0.10 mmol/L	0.07 (0.04-0.09)[a]	0.07 (0.05-0.11)[ac]	0.08 (0.06-0.12)[bc]	0.09 (0.05-0.16)[b]	<.001	0.09 (0.06-0.16)	0.07 (0.05-0.10)	<.001
Glutamine	0.64-1.02 mmol/L	0.67 (0.53-0.97)	0.60 (0.38-0.76)	0.59 (0.44-077)	0.64 (0.47-0.85)	>.05	0.64 (0.47-0.85)	0.72 (0.39-0.83)	>.05
Total BCAA	0.24-0.52 mmol/L	0.38 (0.25-0.54)[a]	0.49 (0.29-1.89)[b]	0.40 (0.26-0.80)[ab]	0.44 (0.27-0.85)[a]	<.01	0.44 (0.27-0.59)	0.37 (0.26-0.81)	>.05
Leucine	0.08-0.19 mmol/L	0.13 (0.07-0.18)	0.16 (0.08-0.72)	0.13 (0.08-0.24)	0.15 (0.09-0.31)	>.05	0.14 (0.09-0.22)	0.13 (0.07-0.27)	>.05
Isoleucine	0.04-0.09 mmol/L	0.07 (0.05-0.10)[a]	0.09 (0.04-0.16)[b]	0.07 (0.05-0.15)[ab]	0.08 (0.04-0.14)[ab]	<.01	0.07 (0.04-0.12)	0.06 (0.05-0.13)	>.05
Valine	0.11-0.25 mmol/L	0.19 (0.12-0.26)[a]	0.25 (0.17-1.09)[b]	0.20 (0.14-0.41)[a]	0.21 (0.11-0.45)[a]	<.001	0.20 (0.11-0.28)	0.19 (0.12-0.42)	>.05
Alanine	0.22-0.60 mmol/L	0.27 (0.19-0.45)[a]	0.44 (0.33-0.74)[b]	0.31 (0.15-0.48)[a]	0.28 (0.13-0.94)[a]	<.001	0.27 (0.13-0.94)	0.34 (0.20-0.59)	>.05
Glycine	0.13-0.45 mmol/L	0.40 (0.11-0.62)	0.37 (0.05-1.09)	0.37 (0.03-0.50)	0.37 (0.22-0.82)	>.05	0.36 (0.22-0.82)	0.36 (0.22-0.70)	>.05
Alanine/BCAA	0.6-1.6	0.7 (0.5-1.2)[ab]	0.9 (0.2-1.2)[b]	0.7 (0.4-1.3)[ac]	0.6 (0.2-1.7)[a]	<.05	0.6 (0.2-1.7)	0.9 (0.3-1.5)	>.05
Alanine/Valine	1.2-3.5	1.5 (1.0-2.8)	1.8 (0.3-2.5)	1.3 (0.9-2.7)	1.3 (0.5-3.3)	>.05	1.3 (0.5-3.3)	1.8 (0.7-2.9)	>.05
BCAA/Tyrosine	3.8-9.2	5.9 (3.8-8.7)	6.2 (4.7-24.6)	6.6 (4.2-10.9)	6.4 (3.8-13.1)	>.05	6.1 (3.8-9.8)	6.2 (4.1-9.9)	>.05
Phenylalanine/Tyrosine	0.5-1.0	0.7 (0.5-1.0)[a]	0.8 (0.6-1.0)[ac]	0.9 (0.5-1.4)[c]	1.0 (0.7-2.3)[b]	<.001	1.0 (0.8-2.3)	0.7 (0.4-1.0)	<.001
Glycine/Valine	0.7-3.0	2.1 (0.8-3.7)[a]	1.5 (0.2-2.4)[c]	1.6 (0.1-3.2)[bc]	1.7 (1.0-4.5)[ab]	<.01	1.7 (1.1-4.5)	1.9 (1.2-3.3)	>.05
Glycine/BCAA	0.3-1.5	1.1 (0.3-1.8)[a]	0.7 (0.1-1.3)[b]	0.8 (0.1-1.6)[ab]	0.8 (0.5-1.9)[ab]	<.05	0.8 (0.5-1.9)	0.9 (0.6-1.6)	>.05
**Fatty acids**, median (range)									
Total fatty acids	9.7-21.0 mmol/L	15.0 (10.2-20.3)[a]	23.8 (11.8-35.9)[c]	17.6 (10.8-27.7)[ac]	11.6 (6.7-24.2)[b]	<.001	11.7 (6.7-24.2)	18.2 (9.5-29.8)	<.05
Polyunsaturated fatty acids	4.7-11.1 mmol/L	7.7 (5.0-11.1)[a]	12.0 (5.7-18.4)[c]	8.5 (5.2-12.6)[ac]	5.8 (3.5-10.9)[b]	<.001	5.8 (3.5-10.9)	8.5 (4.8-14.3)	<.05
Omega-6 fatty acids	4.1-9.8 mmol/L	7.0 (4.5-9.7)[a]	11.1 (5.2-16.3)[c]	7.7 (4.7-11.6)[a]	5.3 (3.0-9.7)[b]	<.001	5.3 (3.0-9.7)	7.6 (4.4-13.3)	<.01
Arachidonic acid	1.4-3.6 mmol/L	2.4 (1.5-4.0)[a]	4.1 (1.5-5.8)[c]	2.8 (1.4-4.1)[a]	1.9 (0.8-3.5)[b]	<.001	1.8 (0.8-2.9)	2.7 (1.4-4.3)	>.05
Linoleic acid	2.5-5.9 mmol/L	4.2 (2.7-5.8)[a]	6.7 (3.4-10.1)[c]	4.8 (3.1-7.5)[ac]	2.9 (0.1-6.4)[b]	<.001	2.9 (0.1-6.4)	5.1 (2.4-8.7)	<.001
Omega-3 fatty acids	0.4-1.6 mmol/L	0.8 (0.3-1.4)	0.7 (0.2-2.1)	0.8 (0.4-1.4)	0.6 (0.3-1.2)	>.05	0.6 (0.3-1.2)	0.8 (0.3-1.3)	>.05
Docosapentaenoic acid	0.1-0.4 mmol/L	0.2 (0.1-0.4)[a]	0.3 (0.1-0.6)[a]	0.2 (0.1-0.3)[a]	0.1 (0.0-0.3)[b]	<.001	0.1 (0.0-0.3)	0.2 (0.0-0.3)	>.05
Docosahexaenoic acid	0.1-0.7 mmol/L	0.2 (0.0-0.6)	0.3 (0.0-0.5)	0.3 (0.1-0.6)	0.3 (0.1-0.5)	>.05	0.3 (0.1-0.5)	0.2 (0.0-0.4)	>.05
Oleic acid	1.3-2.8 mmol/L	2.0 (1.3-2.9)[a]	2.9 (1.6-5.0)[c]	2.4 (1.4-4.5)[c]	1.3 (0.7-4.1)[b]	<.001	1.4 (0.7-4.1)	2.5 (1.0-5.1)	<.01

Parameter	Reference interval*	CONT‡	HYPER$_U$‡	HYPER$_T$‡	HYPO$_U$‡	*p* value‡	HYPO$_U$¥	HYPO$_T$¥	*p* value¥
**Fatty acids**, median (range)									
Saturated fatty acids	3.6-7.4 mmol/L	5.5 (3.9-7.1)[a]	9.0 (4.5-12.6)[c]	6.6 (4.2-10.6)[c]	4.5 (2.6-9.1)[b]	<.001	4.6 (2.6-9.1)	6.8 (3.6-10.9)	<.01
Stearic acid	1.7-3.8 mmol/L	2.7 (1.8-3.6)[a]	4.5 (2.1-6.2)[c]	3.2 (1.9-5.0)[c]	2.1 (1.2-4.1)[b]	<.001	2.1 (1.2-4.1)	3.4 (1.7-5.4)	<.01
Palmitic acid	1.8-3.6 mmol/L	2.8 (2.0-3.5)[a]	4.3 (2.4-6.4)[c]	3.5 (2.3-5.6)[c]	2.3 (1.4-5.0)[b]	<.001	2.3 (1.4-5.0)	3.4 (1.9-5.7)	<.01
Polyunsaturated fatty acids % of total fatty acids	47.5-54.9%	51.2 (47.1-55.5)	51.2 (44.0-54.7)	49.3 (39.2-52.4)	50.6 (45.1-55.3)	>.05	50.6 (45.1-53.3)	49.0 (42.2-52.2)	>.05
Omega-6 fatty acids % of total fatty acids	41.6-47.5%	45.5 (41.6-49.7)[ac]	47.4 (41.2-50.8)[c]	44.9 (32.6-47.7)[ab]	44.9 (38.1-50.3)[ab]	<.05	45.0 (38.1-47.8)	44.8 (38.5-47.5)	>.05
Arachidonic acid % of total fatty acids	13.2-20.1%	16.4 (10.9-23.9)	17.5 (10.3-23.2)	15.2 (8.5-18.9)	17.0 (10.3-22.7)	>.05	16.6 (10.3-22.7)	13.9 (10.1-20.1)	>.05
Linoleic acid % of total fatty acids	24.1-28.6%	27.7 (24.8-30.4)[a]	28.0 (24.3-31.6)[a]	27.3 (21.7-30.1)[a]	25.9 (1.3-28.5)[b]	<.001	25.5 (1.3-27.6)	27.9 (24.6-30.2)	<.05
Omega-3 fatty acids % of total fatty acids	3.4-10.4%	5.4 (2.1-8.6)[a]	3.3 (1.1-6.6)[b]	4.4 (2.7-7.2)[ab]	5.8 (3.3-9.9)[a]	<.01	6.1 (3.3-9.9)	4.7 (2.0-7.3)	<.05
Docosapentaenoic acid % of total fatty acids	1.1-2.0%	1.4 (0.8-2.1)	1.2 (0.6-1.8)	1.2 (0.6-1.7)	1.1 (0.0-1.8)	>.05	1.2 (0.0-1.8)	1.0 (0.3-1.5)	>.05
Docosahexaenoic acid % of total fatty acids	0.7-4.8%	1.7 (0.1-3.5)[a]	1.2 (0.0-2.1)[c]	1.7 (0.5-2.5)[ac]	2.3 (0.7-4.5)[b]	<.001	2.3 (1.1-4.5)	1.2 (0.0-2.3)	<.001
Saturated fatty acids % of total fatty acids	33.8-37.9%	36.3 (34.6-38.5)[a]	36.9 (34.0-39.8)[ac]	37.5 (36.1-40.6)[c]	38.0 (34.7-42.0)[b]	<.001	37.9 (34.7-42.0)	37.7 (35.4-39.9)	>.05
Oleic acid % of total fatty acids	10.7-15.1%	12.5 (8.9-15.7)[a]	12.4 (9.9-16.2)[ab]	13.6 (11.1-20.2)[ac]	11.4 (7.8-17.1)[b]	<.05	11.4 (9.2-17.1)	13.7 (10.5-18.9)	>.05
Stearic acid % of total fatty acids	17.3-19.4%	18.0 (16.1-18.9)	18.6 (17.3-19.8)	18.1 (17.1-18.9)	18.3 (15.8-20.4)	>.05	18.2 (15.8-20.3)	18.3 (17.0-19.3)	>.05
Palmitic acid % of total fatty acids	15.9-19.5%	18.5 (16.7-19.9)[a]	18.0 (16.5-20.6)[a]	19.1 (18.0-23.3)[b]	19.7 (16.5-22.8)[b]	<.001	19.6 (17.1-22.8)	19.6 (17.3-21.4)	>.05
Omega-6/Omega-3 fatty acids	4.2-13.4	8.7 (5.3-22.7)[a]	14.5 (6.9-42.8)[b]	10.4 (4.9-15.7)[a]	7.5 (3.8-14.6)[a]	<.01	7.5 (3.8-14.6)	9.6 (5.7-22.8)	>.05
**Glycolysis related metabolites**, median (range)									
Glucose	4.4-6.8 mmol/L	4.8 (3.5-5.7)	5.4 (2.5-22.4)	4.5 (0.9-6.3)	4.4 (2.4-14.2)	>.05	4.4 (2.4-14.2)	4.7 (3.9-6.9)	>.05
Lactate	1.1-3.6 mmol/L	1.6 (0.8-4.2)[a]	3.7 (2.2-14.4)[b]	2.0 (1.1-5.4)[a]	2.0 (0.7-3.9)[a]	<.001	2.0 (1.0-3.9)	1.9 (1.0-5.8)	>.05
Pyruvate	0.01-0.11 mmol/L	0.04 (0.02-0.10)[a]	0.07 (0.03-0.21)[b]	0.04 (0.02-0.10)[a]	0.04 (0.01-0.14)[a]	<.01	0.03 (0.01-0.14)	0.05 (0.02-0.18)	>.05
Acetate	0.02-0.04 mmol/L	0.02 (0.02-0.04)[a]	0.04 (0.03-0.08)[c]	0.03 (0.02-0.09)[bc]	0.03 (0.02-0.08)[b]	<.001	0.03 (0.02-0.07)	0.02 (0.01-0.03)	>.05
Citrate	0.06-0.12 mmol/L	0.07 (0.05-0.09)[a]	0.08 (0.05-0.13)[b]	0.07 (0.06-0.12)[ab]	0.08 (0.05-0.43)[b]	<.05	0.09 (0.06-0.43)	0.09 (0.06-0.14)	>.05
**Inflammation marker**, median (range)									
GlycA	0.60-1.03 mmol/L	0.66 (0.50-0.99)[a]	1.33 (0.74-2.28)[c]	1.08 (0.66-1.48)[bc]	0.82 (0.53-2.23)[b]	<.001	0.82 (0.53-2.23)	1.13 (0.71-1.85)	>.05

Parameter	Reference interval*	CONT‡	HYPER$_U$‡	HYPER$_T$‡	HYPO$_U$‡	*p* value‡	HYPO$_U$¥	HYPO$_T$¥	*p* value¥
**Fluid balance**, median (range)									
Albumin	25-32 g/L	28 (24-31)^{ac}	30 (23-34)^{c}	27 (22-30)^{ab}	25 (20-33)^{ab}	<.05	25 (20-33)	30 (22-35)	<.05
Creatinine	32-103 μmol/L	60 (25-76)^{ab}	46 (12-77)^{c}	58 (24-164)^{ac}	76 (25-366)^{b}	<.001	62 (27-366)	60 (22-98)	>.05
**Triglycerides**, median (range)									
Total triglycerides	0.19-1.00 mmol/L	0.38 (0.05-0.87)^{a}	0.82 (0.30-2.61)^{b}	0.71 (0.22-1.58)^{b}	0.35 (0.07-3.72)^{a}	<.001	0.38 (0.07-3.72)	0.67 (0.20-2.26)	>.05
VLDL triglycerides	0.00-0.70 mmol/L	0.12 (0.00-0.53)^{a}	0.59 (0.12-2.14)^{b}	0.46 (0.04-1.24)^{b}	0.12 (0.00-2.98)^{a}	<.001	0.13 (0.01-2.98)	0.42 (0.05-1.68)	>.05
LDL triglycerides	0.13-0.31 mmol/L	0.22 (0.04-0.34)	0.20 (0.00-0.38)	0.23 (0.06-0.36)	0.19 (0.03-0.51)	>.05	0.18 (0.03-0.51)	0.23 (0.10-0.41)	>.05
HDL triglycerides	0.00-0.08 mmol/L	0.03 (0.01-0.08)^{a}	0.08 (0.03-0.15)^{b}	0.07 (0.02-0.15)^{b}	0.03 (0.00-0.23)^{a}	<.001	0.03 (0.00-0.23)	0.07 (0.01-0.17)	>.05
**Cholesterol**, median (range)									
Total cholesterol	3.6-10.3 mmol/L	6.4 (3.8-10.0)^{a}	10.1 (4.2-17.0)^{c}	6.8 (3.9-11.4)^{a}	4.2 (2.2-8.5)^{b}	<.001	3.9 (2.2-6.6)	6.5 (3.4-13.2)	<.01
Esterified cholesterol	2.9-8.1 mmol/L	5.2 (3.2-8.0)^{a}	7.9 (3.4-13.2)^{c}	5.4 (3.2-8.8)^{a}	3.3 (1.9-6.9)^{b}	<.001	3.3 (1.9-5.2)	5.3 (2.9-10.4)	<.01
Free cholesterol	0.6-2.2 mmol/L	1.2 (0.6-2.1)^{a}	2.1 (0.8-3.8)^{c}	1.4 (0.7-2.6)^{a}	0.8 (0.4-1.7)^{b}	<.001	0.8 (0.4-1.4)	1.3 (0.5-2.9)	<.01
VLDL cholesterol	0.0-0.3 mmol/L	0.1 (0.0-0.4)^{a}	0.4 (0.1-1.9)^{b}	0.3 (0.1-0.5)^{b}	0.1 (0.1-0.8)^{a}	<.001	0.1 (0.1-0.8)	0.2 (0.1-0.9)	>.05
LDL cholesterol	0.3-2.3mmol/L	1.0 (0.3-2.8)^{a}	2.5 (0.5-7.5)^{c}	1.2 (0.2-4.0)^{a}	0.6 (0.2-2.0)^{b}	<.001	0.6 (0.4-1.9)	1.0 (0.4-4.4)	>.05
HDL cholesterol	3.2-7.9 mmol/L	5.3 (3.5-7.4)^{a}	7.2 (3.7-9.8)^{c}	5.5 (3.2-7.7)^{a}	3.6 (1.8-6.3)^{b}	<.001	3.3 (1.8-5.1)	5.3 (3.0-9.8)	<.01
**Total lipids**, median (range)									
VLDL lipids	0.1-1.2 mmol/L	0.3 (0.1-1.0)^{a}	1.3 (0.4-5.0)^{b}	0.9 (0.2-2.3)^{b}	0.3 (0.1-4.6)^{a}	<.001	0.3 (0.1-4.6)	0.8 (0.2-3.0)	>.05
LDL lipids	0.7-3.7 mmol/L	1.8 (0.7-4.4)^{a}	4.2 (1.2-11.4)^{c}	2.1 (0.8-6.1)^{a}	1.0 (0.6-3.3)^{b}	<.001	1.0 (0.6-3.1)	1.8 (0.7-6.8)	>.05
HDL lipids	6.9-15.1 mmol/L	10.9 (7.5-14.4)^{a}	15.0 (8.2-18.7)^{c}	11.6 (7.1-15.6)^{a}	7.7 (4.4-12.8)^{b}	<.001	7.5 (4.4-10.8)	11.2 (6.8-18.5)	<.001
**Particle concentrations**, median (range)									
VLDL particles	0.01-0.05 μmol/L	0.03 (0.01-0.06)^{a}	0.08 (0.02-0.26)^{b}	0.05 (0.02-0.10)^{b}	0.03 (0.02-0.14)^{a}	<.001	0.03 (0.02-0.14)	0.05 (0.02-0.14)	>.05
LDL particles	0.24-1.30 μmol/L	0.65 (0.26-1.66)^{a}	1.52 (0.38-4.12)^{c}	0.77 (0.27-2.32)^{a}	0.37 (0.21-1.19)^{b}	<.001	0.39 (0.25-1.12)	0.68 (0.28-2.41)	>.05
HDL particles	0.03-0.06 mmol/L	0.04 (0.03-0.05)^{a}	0.05 (0.02-0.06)^{c}	0.04 (0.03-0.05)^{a}	0.03 (0.01-0.05)^{b}	<.001	0.03 (0.01-0.04)	0.04 (0.03-0.06)	<.01
**Average diameter of particles**, median (range)									
VLDL size	35.2-43.8 nm	38.1 (35.0-43.1)^{a}	39.7 (37.9-46.6)^{bc}	40.9 (35.4-45.0)^{c}	38.7 (35.2-45.3)^{ab}	<.01	39.4 (35.2-45.3)	40.2 (36.6-45.6)	>.05
LDL size	22.2-23.5 nm	22.6 (22.2-23.5)^{a}	22.7 (22.3-23.4)^{a}	22.6 (22.3-23.5)^{a}	23.2 (22.1-23.9)^{b}	<.001	23.2 (22.4-23.9)	22.7 (22.1-23.7)	<.05
HDL size	10.1-10.7 nm	10.6 (10.2-11.0)^{a}	10.7 (10.2-11.3)^{a}	10.6 (10.3-11.3)^{a}	10.3 (10.1-11.2)^{b}	<.01	10.3 (10.1-11.2)	10.6 (10.1-11.0)	>.05

Parameter	Reference interval*	CONT‡	HYPER$_U$‡	HYPER$_T$‡	HYPO$_U$‡	p value‡	HYPO$_U$¥	HYPO$_T$¥	p value¥
**Lipoprotein subclasses**, median (range)									
L-HDL cholesterol	2.3-4.4 mmol/L	3.1 (2.2-3.8)[a]	3.7 (1.9-4.7)[a]	3.2 (2.1-4.0)[a]	2.3 (1.3-3.8)[b]	<.001	2.1 (1.3-3.3)	3.2 (2.2-4.4)	<.01
L-HDL esterified cholesterol	2.0-3.8 mmol/L	2.7 (1.9-3.3)[a]	3.1 (1.5-4.0)[a]	2.7 (1.8-3.3)[a]	2.0 (1.1-3.3)[b]	<.001	1.8 (1.1-2.9)	2.7 (1.9-3.7)	<.01
L-HDL free cholesterol	0.3-0.6 mmol/L	0.4 (0.3-0.6)[a]	0.6 (0.3-0.7)[c]	0.5 (0.3-0.7)[a]	0.3 (0.2-0.5)[b]	<.001	0.3 (0.2-0.4)	0.4 (0.3-0.7)	<.01
L-HDL lipids	5.0-8.3 mmol/L	6.1 (4.7-7.5)[a]	7.6 (4.1-9.6)[c]	6.5 (4.4-8.2)[ac]	4.9 (3.0-7.5)[b]	<.001	4.4 (3.0-6.9)	6.7 (4.6-8.1)	<.01
L-HDL particles	0.02-0.03 mmol/L	0.02 (0.02-0.03)[a]	0.03 (0.01-0.04)[ac]	0.02 (0.02-0.03)[a]	0.02 (0.01-0.03)[b]	<.001	0.02 (0.01-0.03)	0.02 (0.02-0.03)	<.01
L-HDL phospholipids	2.48-3.94 mmol/L	3.08 (2.42-3.74)[a]	3.86 (2.22-4.98)[c]	3.28 (2.31-4.46)[a]	2.62 (1.72-3.76)[b]	<.001	2.50 (1.72-3.49)	3.32 (2.31-4.14)	<.01
L-HDL triglycerides	0.00-0.03 mmol/L	0.01 (0.00-0.03)[a]	0.03 (0.01-0.05)[b]	0.02 (0.01-0.05)[b]	0.01 (0.00-0.06)[a]	<.001	0.01 (0.00-0.06)	0.03 (0.01-0.06)	<.05
L-LDL cholesterol	0.07-0.68 mmol/L	0.31 (0.08-1.03)[a]	0.89 (0.14-3.35)[b]	0.38 (0.09-1.29)[a]	0.26 (0.06-0.72)[a]	<.001	0.28 (0.16-0.59)	0.35 (0.06-1.72)	>.05
L-LDL esterified cholesterol	0.03-0.49 mmol/L	0.20 (0.02-0.76)[a]	0.65 (0.06-2.51)[b]	0.27 (0.03-0.97)[a]	0.18 (0.02-0.52)[a]	<.001	0.20 (0.08-0.42)	0.24 (0.01-1.27)	>.05
L-LDL free cholesterol	0.04-0.20 mmol/L	0.11 (0.05-0.27)[a]	0.24 (0.04-0.84)[b]	0.11 (0.07-0.32)[a]	0.09 (0.04-0.20)[a]	<.001	0.09 (0.06-0.16)	0.11 (0.05-0.45)	>.05
L-LDL lipids	0.3-1.2 mmol/L	0.7 (0.4-1.6)[a]	1.5 (0.5-5.0)[b]	0.8 (0.4-1.9)[a]	0.5 (0.3-1.3)[a]	<.001	0.6 (0.3-1.0)	0.7 (0.3-2.6)	>.05
L-LDL particles	0.09-0.37 μmol/L	0.22 (0.11-0.54)[ac]	0.49 (0.13-1.52)[b]	0.23 (0.13-0.64)[c]	0.17 (0.08-0.39)[a]	<.001	0.18 (0.11-0.32)	0.23 (0.10-0.84)	>.05
L-LDL phospholipids	0.08-0.32 mmol/L	0.20 (0.10-0.51)[ac]	0.48 (0.11-1.50)[b]	0.21 (0.15-0.59)[c]	0.17 (0.08-0.37)[a]	<.001	0.17 (0.10-0.30)	0.23 (0.10-0.82)	>.05
L-LDL triglycerides	0.10-0.24 mmol/L	0.17 (0.01-0.28)	0.16 (0.00-0.31)	0.17 (0.05-0.27)	0.15 (0.02-0.37)	>.05	0.15 (0.02-0.37)	0.17 (0.08-0.29)	>.05
L-VLDL cholesterol	0.00-0.13 mmol/L	0.04 (0.00-0.13)[a]	0.16 (0.02-0.72)[b]	0.09 (0.01-0.22)[b]	0.04 (0.01-0.35)[a]	<.001	0.05 (0.01-0.35)	0.09 (0.03-0.35)	>.05
L-VLDL esterified cholesterol	0.00-0.06 mmol/L	0.02 (0.00-0.08)[a]	0.08 (0.01-0.39)[c]	0.03 (0.00-0.07)[b]	0.02 (0.00-0.09)[ab]	<.001	0.02 (0.00-0.09)	0.03 (0.01-0.17)	>.05
L-VLDL free cholesterol	0.00-0.08 mmol/L	0.02 (0.00-0.06)[a]	0.08 (0.02-0.33)[b]	0.06 (0.00-0.16)[b]	0.02 (0.01-0.26)[a]	<.001	0.02 (0.01-0.26)	0.04 (0.01-0.22)	>.05
L-VLDL lipids	0.0-0.6 mmol/L	0.1 (0.0-0.5)[a]	0.6 (0.1-2.2)[b]	0.4 (0.0-1.0)[b]	0.1 (0.0-2.1)[a]	<.001	0.1 (0.0-2.1)	0.4 (0.0-1.3)	>.05
L-VLDL particles	0.00-0.02 μmol/L	0.00 (0.00-0.01)[a]	0.02 (0.00-0.06)[b]	0.01 (0.00-0.03)[b]	0.00 (0.00-0.05)[a]	<.001	0.00 (0.00-0.05)	0.01 (0.00-0.04)	>.05
L-VLDL phospholipids	0.00-0.11 mmol/L	0.02 (0.00-0.08)[a]	0.11 (0.01-0.44)[b]	0.07 (0.00-0.22)[b]	0.02 (0.00-0.35)[a]	<.001	0.02 (0.00-0.35)	0.05 (0.01-0.30)	>.05
L-VLDL triglycerides	0.01-0.42 mmol/L	0.04 (0.00-0.29)[a]	0.32 (0.04-1.08)[b]	0.23 (0.00-0.61)[b]	0.04 (0.00-1.37)[a]	<.001	0.05 (0.00-1.37)	0.19 (0.00-0.80)	>.05
S-HDL lipids	1.3-2.2 mmol/L	1.5 (1.0-1.9)[a]	1.9 (0.8-2.6)[c]	1.5 (0.8-2.0)[a]	1.2 (0.5-1.9)[b]	<.001	1.1 (0.5-1.8)	1.6 (0.9-2.0)	<.05
S-HDL cholesterol	0.5-1.0 mmol/L	0.6 (0.4-0.8)[a]	0.8 (0.3-1.1)[a]	0.6 (0.3-0.8)[a]	0.5 (0.2-0.8)[b]	<.001	0.5 (0.2-0.8)	0.7 (0.4-0.9)	<.05
S-HDL esterified cholesterol	0.4-0.8 mmol/L	0.5 (0.3-0.7)[a]	0.6 (0.2-0.9)[a]	0.5 (0.2-0.7)[ab]	0.4 (0.1-0.6)[b]	<.05	0.4 (0.1-0.6)	0.5 (0.3-0.7)	<.05
S-HDL free cholesterol	0.1-0.2 mmol/L	0.1 (0.1-0.2)[a]	0.2 (0.1-0.2)[c]	0.1 (0.1-0.2)[a]	0.1 (0.0-0.2)[b]	<.001	0.1 (0.0-0.1)	0.1 (0.1-0.2)	<.01
S-HDL particles	0.01-0.02 mmol/L	0.01 (0.01-0.02)[a]	0.02 (0.01-0.02)[c]	0.01 (0.01-0.02)[a]	0.01 (0.00-0.02)[b]	<.001	0.01 (0.00-0.02)	0.01 (0.01-0.02)	<.05

Parameter	Reference interval*	CONT‡	HYPER$_{U}$‡	HYPER$_{T}$‡	HYPO$_{U}$‡	*p* value‡	HYPO$_{U}$¥	HYPO$_{T}$¥	*p* value¥
**Lipoprotein subclasses**, median (range)									
S-HDL phospholipids	0.72-1.19 mmol/L	0.82 (0.61-1.08)[a]	1.11 (0.47-1.46)[c]	0.86 (0.49-1.24)[a]	0.71 (0.33-1.10)[b]	<.001	0.61 (0.33-1.02)	0.89 (0.56-1.14)	<.05
S-HDL triglycerides	0.00-0.03 mmol/L	0.01 (0.00-0.04)[a]	0.03 (0.00-0.04)[b]	0.03 (0.00-0.07)[bc]	0.02 (0.00-0.09)[ac]	<.001	0.02 (0.00-0.09)	0.03 (0.00-0.09)	>.05
S-LDL cholesterol	0.16-1.53 mmol/L	0.67 (0.16-1.74)[a]	1.60 (0.28-4.38)[c]	0.77 (0.10-2.68)[a]	0.26 (0.13-1.28)[b]	<.001	0.27 (0.13-1.28)	0.72 (0.16-2.63)	>.05
S-LDL esterified cholesterol	0.10-1.09 mmol/L	0.48 (0.11-1.26)[a]	1.14 (0.20-3.19)[c]	0.57 (0.09-1.94)[a]	0.18 (0.10-0.93)[b]	<.001	0.20 (0.10-0.92)	0.51 (0.11-1.92)	>.05
S-LDL free cholesterol	0.05-0.45 mmol/L	0.19 (0.06-0.49)[a]	0.44 (0.08-1.19)[c]	0.21 (0.02-0.74)[a]	0.07 (0.03-0.37)[b]	<.001	0.07 (0.04-0.37)	0.20 (0.03-0.71)	>.05
S-LDL lipids	0.3-2.4 mmol/L	1.1 (0.3-2.8)[a]	2.6 (0.5-6.9)[c]	1.3 (0.3-4.2)[a]	0.4 (0.2-2.1)[b]	<.001	0.4 (0.2-2.0)	1.2 (0.3-4.2)	>.05
S-LDL particles	0.14-0.96 μmol/L	0.45 (0.13-1.12)[a]	0.97 (0.20-2.69)[c]	0.53 (0.15-1.67)[a]	0.19 (0.11-0.81)[b]	<.001	0.20 (0.11-0.79)	0.48 (0.13-1.58)	>.05
S-LDL phospholipids	0.12-0.84 mmol/L	0.42 (0.12-1.01)[a]	0.91 (0.19-2.49)[c]	0.46 (0.10-1.50)[a]	0.16 (0.07-0.75)[b]	<.001	0.16 (0.07-0.71)	0.44 (0.10-1.53)	>.05
S-LDL triglycerides	0.03-0.07 mmol/L	0.05 (0.03-0.08)	0.05 (0.00-0.09)	0.05 (0.01-0.09)	0.04 (0.01-0.14)	>.05	0.04 (0.01-0.14)	0.06 (0.01-0.12)	>.05
S-VLDL cholesterol	0.02-0.15 mmol/L	0.06 (0.01-0.21)[a]	0.23 (0.04-1.02)[b]	0.08 (0.03-0.18)[a]	0.06 (0.03-0.18)[a]	<.001	0.06 (0.03-0.14)	0.08 (0.03-0.46)	>.05
S-VLDL esterified cholesterol	0.01-0.09 mmol/L	0.04 (0.00-0.15)[a]	0.15 (0.01-0.76)[b]	0.04 (0.00-0.12)[a]	0.04 (0.00-0.13)[a]	<.001	0.04 (0.00-0.08)	0.04 (0.00-0.32)	>.05
S-VLDL free cholesterol	0.01-0.06 mmol/L	0.03 (0.01-0.06)[a]	0.08 (0.02-0.26)[b]	0.04 (0.02-0.09)[b]	0.03 (0.01-0.10)[a]	<.001	0.03 (0.01-0.10)	0.04 (0.02-0.14)	>.05
S-VLDL lipids	0.1-0.4 mmol/L	0.2 (0.1-0.4)[a]	0.5 (0.2-1.8)[b]	0.3 (0.1-0.6)[b]	0.2 (0.1-0.9)[a]	<.001	0.2 (0.1-0.9)	0.3 (0.1-1.0)	>.05
S-VLDL particles	0.01-0.04 μmol/L	0.03 (0.01-0.05)[a]	0.06 (0.02-0.19)[b]	0.04 (0.02-0.07)[b]	0.02 (0.01-0.09)[a]	<.001	0.02 (0.01-0.09)	0.03 (0.01-0.10)	>.05
S-VLDL phospholipids	0.01-0.08 mmol/L	0.05 (0.02-0.09)[a]	0.12 (0.03-0.41)[b]	0.07 (0.03-0.15)[b]	0.04 (0.02-0.17)[a]	<.001	0.04 (0.02-0.17)	0.07 (0.03-0.22)	>.05
S-VLDL triglycerides	0.02-0.16 mmol/L	0.07 (0.00-0.15)[a]	0.14 (0.04-0.53)[b]	0.13 (0.04-0.27)[b]	0.08 (0.00-0.59)[a]	<.001	0.08 (0.01-0.59)	0.12 (0.05-0.41)	>.05
XL-HDL cholesterol	0.2-2.8 mmol/L	1.6 (0.4-3.4)[a]	2.5 (0.8-5.2)[c]	1.8 (0.7-4.2)[a]	0.6 (0.1-2.3)[b]	<.001	0.6 (0.1-2.3)	1.8 (0.2-4.6)	<.05
XL-HDL esterified cholesterol	0.2-2.1 mmol/L	1.3 (0.3-2.6)[a]	1.9 (0.6-4.0)[c]	1.4 (0.6-3.2)[a]	0.5 (0.0-1.8)[b]	<.001	0.4 (0.0-1.8)	1.4 (0.1-3.5)	<.05
XL-HDL free cholesterol	0.1-0.6 mmol/L	0.4 (0.1-0.8)[a]	0.6 (0.1-1.3)[c]	0.4 (0.2-1.0)[ac]	0.1 (0.0-0.6)[b]	<.001	0.1 (0.0-0.6)	0.4 (0.0-1.0)	<.05
XL-HDL lipids	0.6-5.0 mmol/L	3.3 (1.0-6.4)[a]	5.2 (1.7-9.8)[c]	3.4 (1.6-7.7)[a]	1.5 (0.3-4.4)[b]	<.001	1.4 (0.3-4.3)	3.7 (0.5-8.6)	<.01
XL-HDL particles	0.0-5.8 μmol/L	4.4 (1.2-8.5)[a]	6.5 (2.2-12.0)[c]	4.6 (2.2-9.5)[ac]	1.9 (0.2-5.9)[b]	<.001	1.8 (0.2-5.6)	4.5 (0.4-11.2)	<.05
XL-HDL phospholipids	0.33-2.22 mmol/L	1.64 (0.57-2.99)[a]	2.52 (0.96-4.57)[c]	1.67 (0.84-3.46)[a]	0.80 (0.18-2.19)[b]	<.001	0.80 (0.18-2.00)	1.82 (0.34-3.96)	<.01
XL-HDL triglycerides	0.00-0.02 mmol/L	0.01 (0.00-0.02)[a]	0.02 (0.01-0.06)[b]	0.02 (0.00-0.04)[b]	0.01 (0.00-0.08)[a]	<.001	0.01 (0.00-0.08)	0.02 (0.00-0.05)	<.05
XL-VLDL cholesterol	0.00-0.06 mmol/L	0.01 (0.00-0.05)[a]	0.06 (0.01-0.23)[b]	0.05 (0.00-0.17)[b]	0.01 (0.00-0.30)[a]	<.001	0.01 (0.00-0.30)	0.03 (0.00-0.23)	>.05
XL-VLDL esterified cholesterol	0.00-0.03 mmol/L	0.00 (0.00-0.02)[a]	0.03 (0.00-0.12)[b]	0.02 (0.00-0.06)[b]	0.00 (0.00-0.10)[a]	<.001	0.00 (0.00-0.10)	0.01 (0.00-0.08)	>.05
XL-VLDL free cholesterol	0.00-0.05 mmol/L	0.00 (0.00-0.03)[a]	0.02 (0.00-0.11)[b]	0.03 (0.00-0.12)[b]	0.00 (0.00-0.20)[a]	<.001	0.00 (0.00-0.20)	0.01 (0.00-0.15)	>.05

**Parameter**	**Reference interval***	**CONT**‡	**HYPER$_{U}$**‡	**HYPER$_{T}$**‡	**HYPO$_{U}$**‡	***p* value**‡	**HYPO$_{U}$**¥	**HYPO$_{T}$**¥	***p* value**¥
**Lipoprotein subclasses**, median (range)									
XL-VLDL lipids	0.0-0.3 mmol/L	0.0 (0.0-0.2)[a]	0.2 (0.0-0.9)[b]	0.2 (0.0-0.7)[b]	0.0 (0.0-1.6)[a]	<.001	0.0 (0.0-1.6)	0.1 (0.0-0.9)	>.05
XL-VLDL particles	0.000-0.001 μmol/L	0.000 (0.000-0.001)[a]	0.001 (0.000-0.005)[b]	0.001 (0.000-0.005)[b]	0.000 (0.000-0.008)[a]	<.001	0.000 (0.000-0.008)	0.001 (0.000-0.005)	>.05
XL-VLDL phospholipids	0.00-0.05 mmol/L	0.00 (0.00-0.05)[a]	0.03 (0.00-0.16)[b]	0.04 (0.00-0.17)[b]	0.00 (0.00-0.28)[a]	<.001	0.00 (0.00-0.28)	0.02 (0.00-0.21)	>.05
XL-VLDL triglycerides	0.00-0.17 mmol/L	0.01 (0.00-0.13)[a]	0.09 (0.00-0.53)[b]	0.13 (0.00-0.40)[b]	0.01 (0.00-1.03)[a]	<.001	0.01 (0.00-1.03)	0.05 (0.00-0.47)	>.05

Note. *Reference intervals were established in canine serum for dogs of all ages. ‡Groups of unpaired samples (CONT, HYPER$_{U}$, HYPER$_{T}$, HYPO$_{U}$,) were compared by Kruskal-Wallis test adjusted with Bonferroni-correction. The *p*-values are shown in the 7th column. Level of significance was set at $p<.05$. Results with different letter superscripts ([a, b, c]) in the same line are significantly different from each other. ¥23 paired samples of HYPO$_{U}$ and HYPO$_{T}$ were compared by Wilcoxon signed-rank test adjusted with Bonferroni-correction. The *p*-values are shown in the final column. Level of significance was set at $p<.05$.

BCAA—branched chain amino acid, CONT—control group, GlycA—glycoprotein acetyls, HYPO$_{U}$—hypoadrenocorticism untreated, HYPO$_{T}$—hypoadrenocorticism treated, HYPER$_{U}$—hyperadrenocorticism untreated, HYPER$_{T}$—hyperadrenocorticism treated, L-HDL—large high-density lipoprotein, L-LDL—large low-density lipoprotein, L-VLDL—large very-low-density lipoprotein, S-HDL—small high-density lipoprotein, S-LDL—small low-density lipoprotein, S-VLDL—small very-low-density lipoprotein, XL-HDL—extra-large high-density lipoprotein, XL-VLDL—extra-large very-low-density lipoprotein.

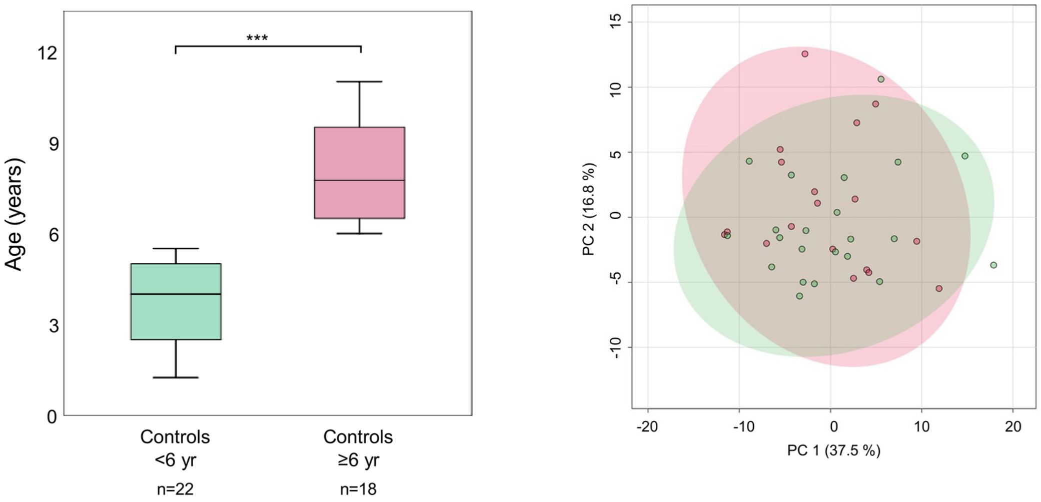

(**a**) (**b**)

Supplementary Figure S1 Comparison of age and metabolomics data of CONT groups subdivided at the age of 6 years into dogs of younger (<6 yrs, n=22) and of older age (≥ 6 yrs, n=18). (**a**) Box plot of age of the subdivided CONT groups. Boxes indicate the lower to upper quartile (25th-75th percentile) and median value. Whiskers extend to minimum and maximum values. Line above the boxes reflects significant difference between the groups using a one-way analysis of variance (ANOVA) of both subdivided CONT, HYPER$_{U}$, HYPER$_{T}$, and HYPO$_{U}$ groups (***p<.001). (**b**) Scores plot of principal component analysis (PCA) showing complete overlap between the samples of the younger (<6 yrs, green) and older adult (≥6 yrs, red) control dogs based on the serum metabolomics data. Shaded circles represent 95% confidence intervals, while colored dots illustrate individual samples. The axes are labelled by the first and second principal component (PC 1 and 2, respectively) and percentage of variance of the data explained by that principal component is shown in parentheses. In addition, univariate comparison of metabolomics data with Kruskal Wallis test adjusted by Bonferroni correction of the HYPER$_{U}$, HYPER$_{T}$, HYPO$_{U}$, and both subdivided CONT groups showed no significant differences between the two age-dependent CONT subgroups, and thus the younger and older adult control dogs were combined to one CONT group.

Note: ANOVA — one-way analysis of variance, CONT—control group, HYPO$_{U}$—hypoadrenocorticism untreated, HYPO$_{T}$—hypoadrenocorticism treated, HYPER$_{U}$—hyperadrenocorticism untreated, HYPER$_{T}$—hyperadrenocorticism treated, PCA – principal component analysis, yrs – years.

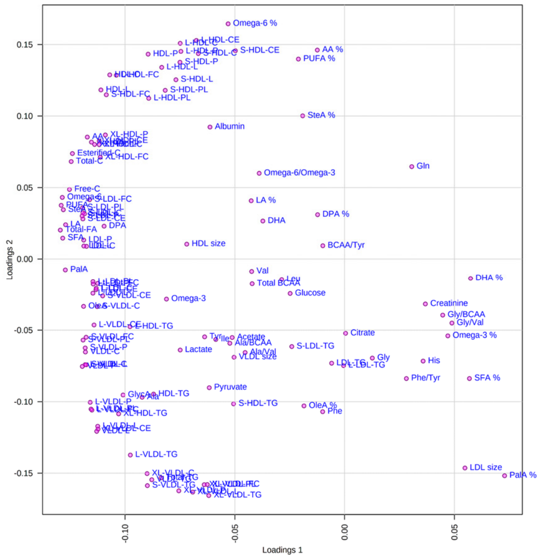

(**a**)

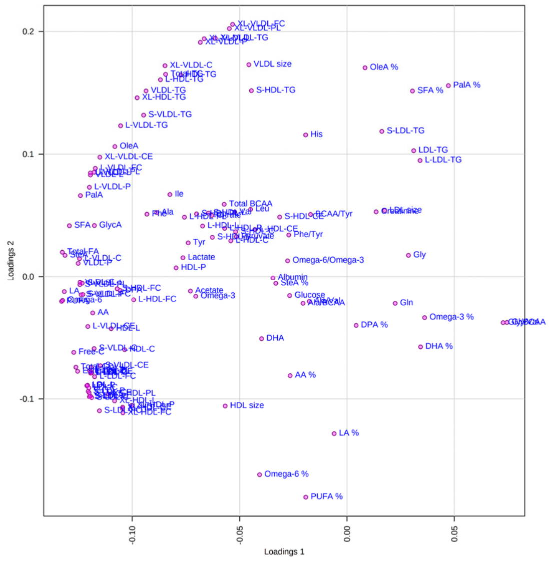

(**b**)

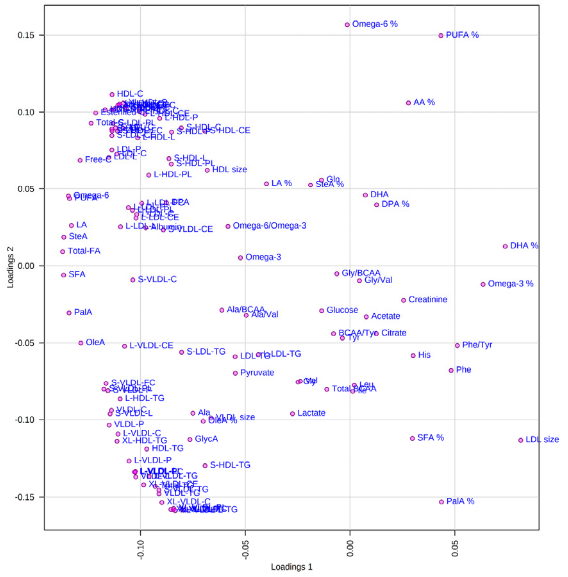

(**c**)

Supplementary Figure S2 Loadings plot of principal component analysis based on metabolomics data between serum samples (**a**) of dogs in the groups of CONT (n=40), HYPERU (n=27), and HYPOU (n=35); (**b**) HYPERU (n=27), HYPERT (n=28), and CONT (n=40); (**c**) HYPOU (n=35), HYPOT (n=23), and CONT (n=40).

Note. AA—arachidonic acid, Ala—alanine, BCAA—branched chain amino acid, C—cholesterol, CE—esterified cholesterol, CONT—control group, DHA—docosahexaenoic acid, DPA—docosapentaenoic acid, FC—free cholesterol, Gln—glutamine, Gly—glycine, GlycA—glycoprotein acetyls, His—histidine, HYPOU—hypoadrenocorticism untreated, HYPOT—hypoadrenocorticism treated, HYPERU— hyperadrenocorticism untreated, HYPERT—hyperadrenocorticism treated, Ile—Isoleucine, LA—linoleic acid, Leu—leucine, L-HDL—large high-density lipoprotein, L-LDL—large low-density lipoprotein, L-VLDL—large very-low-density lipoprotein, OleA—oleic acid, P—particle, PalA—palmitic acid, Phe—phenylalanine, PL—phospholipids, PUFA—polyunsaturated fatty acids, SFA—saturated fatty acids, S-HDL—small high-density lipoprotein, S-LDL—small low-density lipoprotein, SteA—stearic acid, S-VLDL—small very-low-density lipoprotein, TG—triglycerides, Tyr—tyrosine, Val—valine, XL-HDL—extra-large high-density lipoprotein, XL-VLDL—extra-large very-low-density lipoprotein.

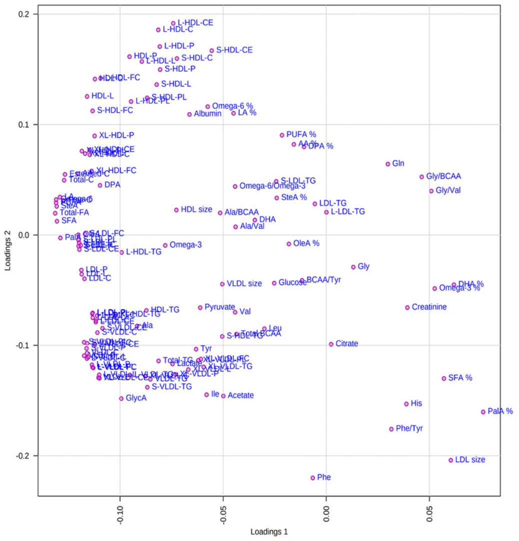

Supplementary Figure S3 Loadings plot of partial least squares-discriminant analysis (PLS-DA) based on metabolomics data between serum samples of dogs in the groups of CONT (n=40), HYPERU (n=27), and HYPOU (n=35).

Note. AA—arachidonic acid, Ala—alanine, BCAA—branched chain amino acid, C—cholesterol, CE—esterified cholesterol, CONT—control group, DHA—docosahexaenoic acid, DPA—docosapentaenoic acid, FC—free cholesterol, Gln—glutamine, Gly—glycine, GlycA—glycoprotein acetyls, His—histidine, HYPOU—hypoadrenocorticism untreated, HYPERU— hyperadrenocorticism untreated, Ile—Isoleucine, LA—linoleic acid, Leu—leucine, L-HDL—large high-density lipoprotein, L-LDL—large low-density lipoprotein, L-VLDL—large very-low-density lipoprotein, OleA—oleic acid, P—particle, PalA—palmitic acid, Phe—phenylalanine, PL—phospholipids, PUFA—polyunsaturated fatty acids, SFA—saturated fatty acids, S-HDL—small high-density lipoprotein, S-LDL—small low-density lipoprotein, SteA—stearic acid, S-VLDL—small very-low-density lipoprotein, TG—triglycerides, Tyr—tyrosine, Val—valine, XL-HDL—extra-large high-density lipoprotein, XL-VLDL—extra-large very-low-density lipoprotein.

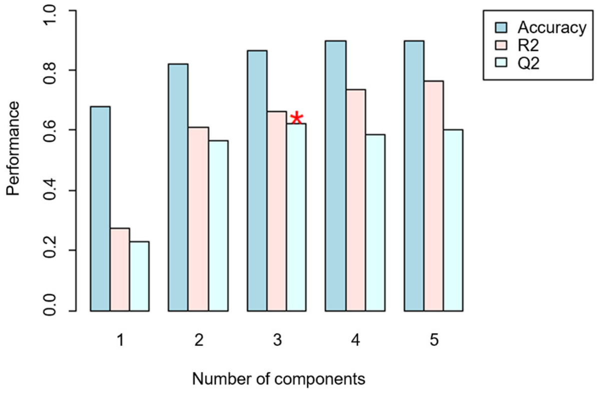

Supplementary Figure S4 Results of the 10-fold cross-validation of the partial least squares-discriminant analysis (PLS-DA) model based on metabolomics data between serum samples of dogs in the groups of CONT (n=40), HYPER$_U$ (n=27), and HYPO$_U$ (n=35) with R^2, Q^2, and accuracy measures based on the number of components. Three components were chosen for the model based on the Q^2 criterion.

Note. CONT—control group, HYPO$_U$—hypoadrenocorticism untreated, HYPER$_U$— hyperadrenocorticism untreated.

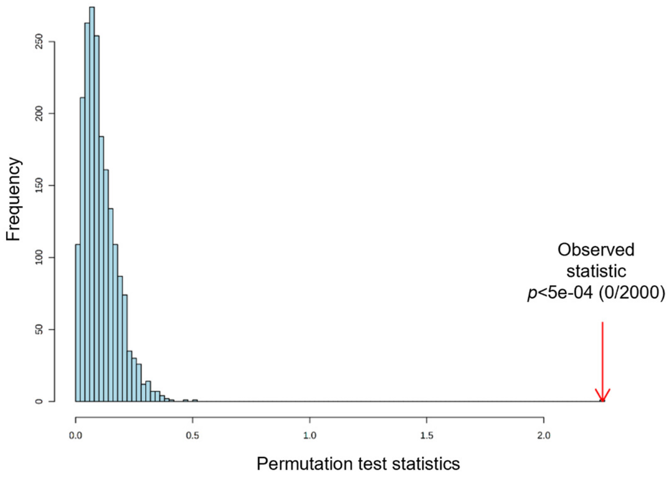

Supplementary Figure S5 Results of a permutation test with 2,000 permutations for the partial least squares-discriminant analysis (PLS-DA) model based on metabolomics data between serum samples of dogs in the groups of CONT (n=40), HYPER$_U$ (n=27), and HYPO$_U$ (n=35). The results that the model is not overfitting the data, $p<5e^{-04}$.

Note. CONT—control group, HYPO$_U$—hypoadrenocorticism untreated, HYPER$_U$— hyperadrenocorticism untreated.

Supplementary Table S2 Different machine learning models classifying groups based solely on metabolomics data from serum samples of dogs in the groups of CONT (n=40), HYPER$_U$ (n=27), and HYPO$_U$ (n=35); HYPER$_U$ (n=27), HYPER$_T$ (n=28), and CONT (n=40); HYPO$_U$ (n=35), HYPO$_T$ (n=23), and CONT (n=40).

Groups	% of correctly classified cases based solely on metabolomics data					
	**Simple Logistic**	**SVM CS**	**MN N Bayes**	**MLP**	**Random Forest**	**KNN**
**CONT, HYPER$_U$, HYPO$_U$**	88.2	78.4	78.4	86.3	81.4	77.5
**CONT, HYPER$_U$, HYPER$_T$**	88.4	85.3	72.6	82.1	72.6	69.5
**CONT, HYPO$_U$, HYPO$_T$**	77.6	69.4	68.4	71.4	75.5	63.3

Note. CONT—control group, HYPO$_U$—hypoadrenocorticism untreated, HYPO$_T$—hypoadrenocorticism treated, HYPER$_U$—hyperadrenocorticism untreated, HYPER$_T$—hyperadrenocorticism treated, KNN—k-nearest neighbors algorithm, MLP—Multilayer Perceptron Classifier, MN N Bayes—multinomial naïve Bayes, SVM CS—Support Vector Machines - Cramer and Singer.

Supplementary Equation S1 Equation of simple logistic regression model of the metabolomics data from serum samples of dogs in the groups of CONT (n=40), HYPERU (n=27), and HYPOU (n=35).

Class CONT: 20.65 + [SFA %] * -0.43 + [Phe] * -46.03 + [GlycA] * -2.04

Class HYPERU: -5.52 + [SteA] * 0.9 + [Lactate] * 0.94

Class HYPOU: -0.85 + [HDL-C] * -0.55 + [His] * 26.29 + [Phe/Tyr] * 1.89

The statistic result for these predictions were (including AUC of the ROC):

Correctly Classified Instances	90	88.2353%
Incorrectly Classified Instances	12	11.7647%
Kappa statistic	0.8209	
Mean absolute error	0.1342	
Root mean squared error	0.2651	
Relative absolute error	30.5483%	
Root relative squared error	56.552%	
Total Number of Instances	102	

Note. CONT—control group, GlycA—glycoprotein acetyls, His—histidine, HYPOU—hypoadrenocorticism untreated, HYPERU—hyperadrenocorticism untreated, HDL-C—high-density lipoprotein cholesterol, Phe—phenylalanine, SFA—saturated fatty acids, SteA—stearic acid, Tyr—tyrosine.

Supplementary Table S3 Detailed accuracy by class for the simple logistic regression model of the metabolomics data from serum samples of dogs in the groups of CONT (n=40), HYPERU (n=27), and HYPOU (n=35).

Class	TP Rate	FP Rate	Precision	Recall	F-Measure	MCC	ROC Area	PRC Area
**CONT**	0.950	0.113	0.844	0.950	0.894	0.823	0.935	0.814
**HYPERU**	0.926	0.040	0.893	0.926	0.909	0.876	0.963	0.914
**HYPOU**	0.771	0.030	0.931	0.771	0.844	0.780	0.899	0.900
**Weighted Average**	0.882	0.065	0.887	0.882	0.881	0.822	0.930	0.870

Note. CONT—control group, HYPOU—hypoadrenocorticism untreated, HYPERU—hyperadrenocorticism untreated.
